# Do Humans Optimally Exploit Redundancy to Control Step Variability in Walking?

**DOI:** 10.1371/journal.pcbi.1000856

**Published:** 2010-07-15

**Authors:** Jonathan B. Dingwell, Joby John, Joseph P. Cusumano

**Affiliations:** 1 Department of Kinesiology, University of Texas, Austin, Texas, United States of America; 2 Department of Engineering Science & Mechanics, Pennsylvania State University, University Park, Pennsylvania, United States of America; University College London, United Kingdom

## Abstract

It is widely accepted that humans and animals minimize energetic cost while walking. While such principles predict average behavior, they do not explain the *variability* observed in walking. For robust performance, walking movements must adapt at each step, not just on average. Here, we propose an analytical framework that reconciles issues of optimality, redundancy, and stochasticity. For human treadmill walking, we defined a goal function to formulate a precise mathematical definition of one possible control strategy: maintain constant speed at each stride. We recorded stride times and stride lengths from healthy subjects walking at five speeds. The specified goal function yielded a decomposition of stride-to-stride variations into new gait variables explicitly related to achieving the hypothesized strategy. Subjects exhibited greatly decreased variability for goal-relevant gait fluctuations directly related to achieving this strategy, but far greater variability for goal-irrelevant fluctuations. More importantly, humans immediately corrected goal-relevant deviations at each successive stride, while allowing goal-irrelevant deviations to persist across multiple strides. To demonstrate that this was not the only strategy people could have used to successfully accomplish the task, we created three surrogate data sets. Each tested a specific alternative hypothesis that subjects used a different strategy that made *no* reference to the hypothesized goal function. Humans did *not* adopt any of these viable alternative strategies. Finally, we developed a sequence of stochastic control models of stride-to-stride variability for walking, based on the Minimum Intervention Principle. We demonstrate that healthy humans are not precisely “optimal,” but instead consistently slightly *over*-correct small deviations in walking speed at each stride. Our results reveal a new governing principle for regulating stride-to-stride fluctuations in human walking that acts independently of, but in parallel with, minimizing energetic cost. Thus, humans exploit task redundancies to achieve robust control while minimizing effort and allowing potentially beneficial motor variability.

## Author Summary

Existing principles used to explain how locomotion is controlled predict average, long-term behavior. However, neuromuscular noise continuously disrupts these movements, presenting a significant challenge for the nervous system. One possibility is that the nervous system must overcome all neuromuscular variability as a constraint limiting performance. Conversely, we show that humans walking on a treadmill exploit redundancy to adjust stepping movements at each stride and maintain performance. This strategy is not required by the task itself, but is predicted by appropriate stochastic control models. Thus, the nervous system simplifies control by strongly regulating goal-relevant fluctuations, while largely ignoring non-essential variations. Properly determining how stochasticity affects control is critical to developing biological models, since neuro-motor fluctuations are intrinsic to these systems. Our work unifies the perspectives of time series analysis researchers, motor coordination researchers, and motor control theorists by providing a single dynamical framework for studying variability in the context of goal-directedness.

## Introduction

Walking is an essential task most people take for granted every day. However, the neural systems that regulate walking perform many complex functions, especially when we walk in unpredictable environments. These systems continuously integrate multiple sensory inputs [1]–[4] and generate motor outputs to coordinate many muscles to achieve efficient, stable, and adaptable locomotion. Establishing the fundamental principles that guide this control is central to understanding how the central nervous system regulates walking.

**Figure 1:**
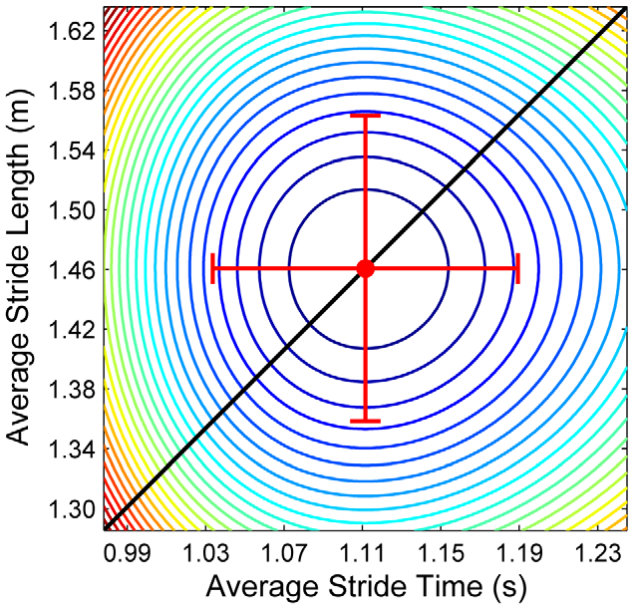
Predicted metabolic cost as a function of average stride length (*L*) and average stride time (*T*). Contour lines represent iso-energy level curves for average energetic cost of transport: i.e., energy expenditure per distance walked per kg of body mass (cal/m/kg). The optimum (i.e., minimal) cost [*T_Opt_*, *L_Opt_*] occurs at the center of the figure. These contours were determined from the empirical equations derived by Zarrugh et al. [7]. Representative results are shown for the nominal gait pattern of one typical subject, after subtracting the metabolic cost of standing [7]. The diagonal black line represents the line of constant speed, *v*, which passes through [*T_Opt_*, *L_Opt_*]. Horizontal and vertical error bars indicate the energetic consequences of ±7% errors in either *T* or *L*, respectively. These are similar in amplitude to ±3 standard deviations in each of these variables, as observed experimentally (Fig. 3D–E), and thus approximate the general range of stride-to-stride variations expected to be observed in these measures. The horizontal and vertical axes are likewise scaled to ±12% change in each variable. These iso-energy contours are nearly isotropic: i.e., relative changes in stride length incur nearly the same energetic cost penalty as comparable relative changes in stride time. (See Supplementary Text S1 for additional details).

The principal idea used to explain how humans and animals regulate walking has been energy cost [5]–[12]. At a given speed, humans choose an average step length and frequency that minimizes energy cost [7], [9], [10], [12]. Small changes in either average stride length or average stride time increase energy cost in humans similarly (Fig. 1, and Supplementary Text S1) [7]. These experimental findings have been supported by multiple computational models [9]–[11], [13], [14]. Such optimality principles have been a major focus for understanding the control of complex movements [15]–[20]. However, these optimization criteria have been used primarily to predict average behavior, not to explain the *variability* ubiquitously observed in movements like walking [21]–[24]. Understanding the nature of this variability may be critical to understanding how humans perform skilled movements [25]–[34]. Most optimization approaches do not address whether the nervous system must *overcome* all variability as a limiting constraint [16], [26], [29], [32], or instead exploits redundancy to *regulate* variability in ways that help maximize task performance [25], [27], [28], [34].

Others have sought to determine how muscles are organized into functional synergies to resolve the inherent redundancy of complex movements [35]–[37]. These efforts likewise characterize average behavior and so also provide few insights into movement variability. Conversely, redundancy gives rise to equifinality: i.e., there are typically an infinite number of ways to perform the same action [25], [38]. Equifinality permits individuals to perform complex tasks reliably and repeatedly while allowing variability in a movement's particulars. This is thought to facilitate adaptability in motor performance [25]. Recent researchers have addressed this issue experimentally using the geometry-based uncontrolled manifold (UCM) approach [39], [40]. A related concept, the minimum intervention principle (MIP) [27], [28], [41] ties these ideas to stochastic optimal control theory and provides a concrete computational framework for predicting precisely how trial-to-trial movement variability arises in redundant motor systems performing tasks with well prescribed goals [18], [27], [28], [41], [42].

During walking, humans need to adapt at *every* step (not just on average) to be able to respond to externally and/or internally generated perturbations [23], [43], [44]. While the neurophysiological mechanisms that enact these responses are well known [1]–[4], the fundamental principles governing adaptation *from stride to stride* remain unknown. Small stride-to-stride fluctuations in gait dynamics are typically assumed to reflect random noise. Indeed, there is ample evidence supporting multiple sensory and motor sources of physiological noise [31], [45]–[48]. However, stride-to-stride variations in gait cycle timing exhibit statistical persistence [22], [49], [50], which has been argued to be “indispensible” to healthy physiological function [51], [52]. Stride intervals become more uncorrelated (i.e., less persistent) in elderly subjects and patients with Huntington's disease [53], but not in patients with peripheral sensory loss [54]. Understanding how stride-to-stride control is enacted therefore requires quantifying not only average magnitudes of variations across strides, but also the specific temporal sequencing of those variations.

Here, we formulate goal functions [25] that give concrete mathematical form to hypotheses on the strategies used to achieve a given task. This provides a unifying framework for reconciling issues of optimality, redundancy, and stochasticity in human walking. Walking on a motor driven treadmill only requires that subjects do not “walk off” either the front or back end of the treadmill. While subjects must, over time, walk at the same average speed as the treadmill, variations in speed due to changes in stride length and/or stride time do occur and can be sustained over several consecutive strides [23], [24], [55], [56]. The main question addressed here is how do people *regulate* these variations?

We present a mathematical definition of a specific hypothesized task strategy [25], [57] with the goal to maintain constant walking speed *at each stride*. This yields a decomposition of stride-to-stride variations into new gait variables explicitly related to achieving this strategy. Time series analyses confirm that humans do indeed adopt this hypothesized strategy. We similarly analyze three alternative strategies that equally achieve the task requirements, but make *no* reference to the hypothesized goal function. Humans do *not* adopt any of these alternatives. Finally, we develop a sequence of stochastic optimal control models of stride-to-stride dynamics to determine if they replicate our observations. These models confirm that healthy humans do carefully regulate their movements explicitly to maintain constant speed at each stride. However, humans do not use strategies that are precisely “optimal” with respect to the employed cost functions, but instead slightly but consistently *over*-correct small deviations in walking speed from each stride to the next.

## Results

**Figure 2:**
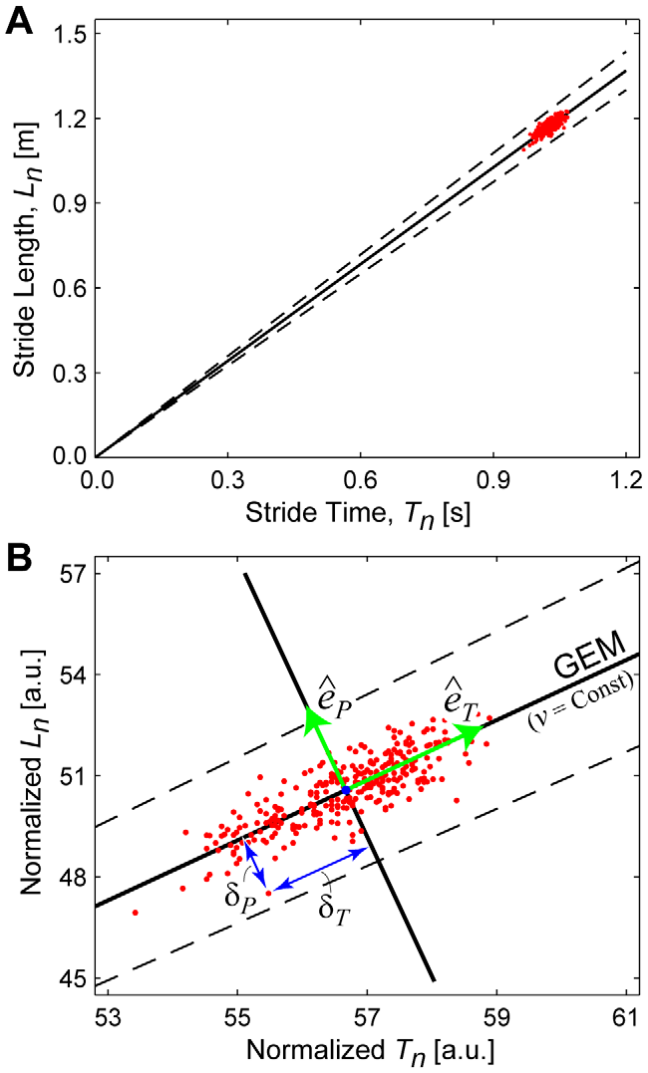
Schematic representation of the goal equivalent manifold (GEM) for walking. (**A**) Example stride time and stride length data. Each dot represents the particular combination of stride length (*L_n_*) and stride time (*T_n_*) for one individual stride. The solid diagonal line defines the set of all combinations of *L_n_* and *T_n_* that achieve the exact same speed, *v*. This line is the Goal Equivalent Manifold (GEM) for walking (Eq. 2) at constant speed *v*. The dashed diagonal lines represent ±5% error in maintaining this constant speed. (**B**) To facilitate the analyses, we non-dimensionalize the data by normalizing the *L_n_* and *T_n_* time series each to unit variance. We then re-define the goal function and the GEM accordingly. We define orthonormal basis vectors, [*ê_T_*, *ê_P_*], aligned tangent to and perpendicular to the GEM, respectively. We then transform the dimensionless *L_n_* and *T_n_* time series into *δ_T_* and *δ_P_* time series of deviations in the *ê_T_* and *ê_P_* directions, respectively, relative to the mean operating point, [T*, L*], along the GEM. Note that the GEM is defined by the average walking speed as set by the treadmill and is therefore independent of how data points representing individual strides are distributed within the [*T_n_*, *L_n_*] plane. The GEM exists prior to and independent of any notions of how people actually control their stride-to-stride movements with respect to it (if at all).

The primary task requirement for walking on a treadmill with belt speed *v* is to not walk off the treadmill. The net change in displacement, relative to the laboratory reference frame, for stride *n* is determined by the stride length, *L_n_*, and stride time, *T_n_*, as 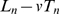. Thus, this task can be mathematically defined by:
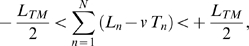
(1)where the summation is the net displacement walked over *N* strides and *L_TM_* is the length of the treadmill belt. A key observation is that *any* sequence of *L_n_* and *T_n_* that satisfies this inequality will successfully accomplish the treadmill walking task. *Many* possible strategies for generating such a sequence of *L_n_* and *T_n_* exist. The simplest strategy can be formulated using the *goal function* [25]:
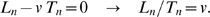
(2)That is, subjects could attempt to maintain constant speed at each stride. This goal function is not a “constraint,” however, because *it is not required* by Eq. (1). It is instead only one possible movement strategy. The solid line in Fig. 2 defines a “Goal Equivalent Manifold” (GEM) [25] containing all [*T_n_*, *L_n_*] pairs that equally satisfy Eq. (2). We hypothesized that humans minimize errors relative to this GEM. Thus, for the present analyses, the relevant stride-to-stride walking dynamics are entirely captured by the impact Poincaré section [58], [59] defined by the [*T_n_*, *L_n_*] plane (Fig. 2).

The hypothesized GEM exists prior to, and independent of, any specific control policy people might adopt to regulate their stepping movements. To determine if humans adopt a strategy that explicitly recognizes this GEM, we defined deviations tangent (*δ_T_*) and perpendicular (*δ_P_*) to it and converted [*T_n_*, *L_n_*] coordinates into GEM-specific [*δ_T_*, *δ_P_*] coordinates (Fig. 2B, Eq. 3). The *δ_T_* deviations are “goal equivalent” because they do *not* affect walking speed, while *δ_P_* deviations are “goal relevant” because they *do*. We therefore hypothesized that subjects would exhibit greater variability in *δ_T_* than in *δ_P_* [25], [27], [28]. We also hypothesized that subjects would not immediately correct deviations along the GEM: i.e., *δ_T_* time series would exhibit statistical persistence [57]. Conversely, we hypothesized that subjects would rapidly correct deviations perpendicular to the GEM: i.e., *δ_P_* time series would exhibit greatly decreased persistence [57], or anti-persistence.

### Primary Dynamical Features of Treadmill Gait

To test GEMs of different location/orientation, subjects walked on a motorized treadmill at each of 5 constant speeds, from 80% to 120% of their preferred walking speed (PWS). Time series of stride times (*T_n_*), stride lengths (*L_n_*), and stride speeds (*S_n_* = *L_n_*/*T_n_*) for all strides within each trial were obtained and analyzed.

**Figure 3:**
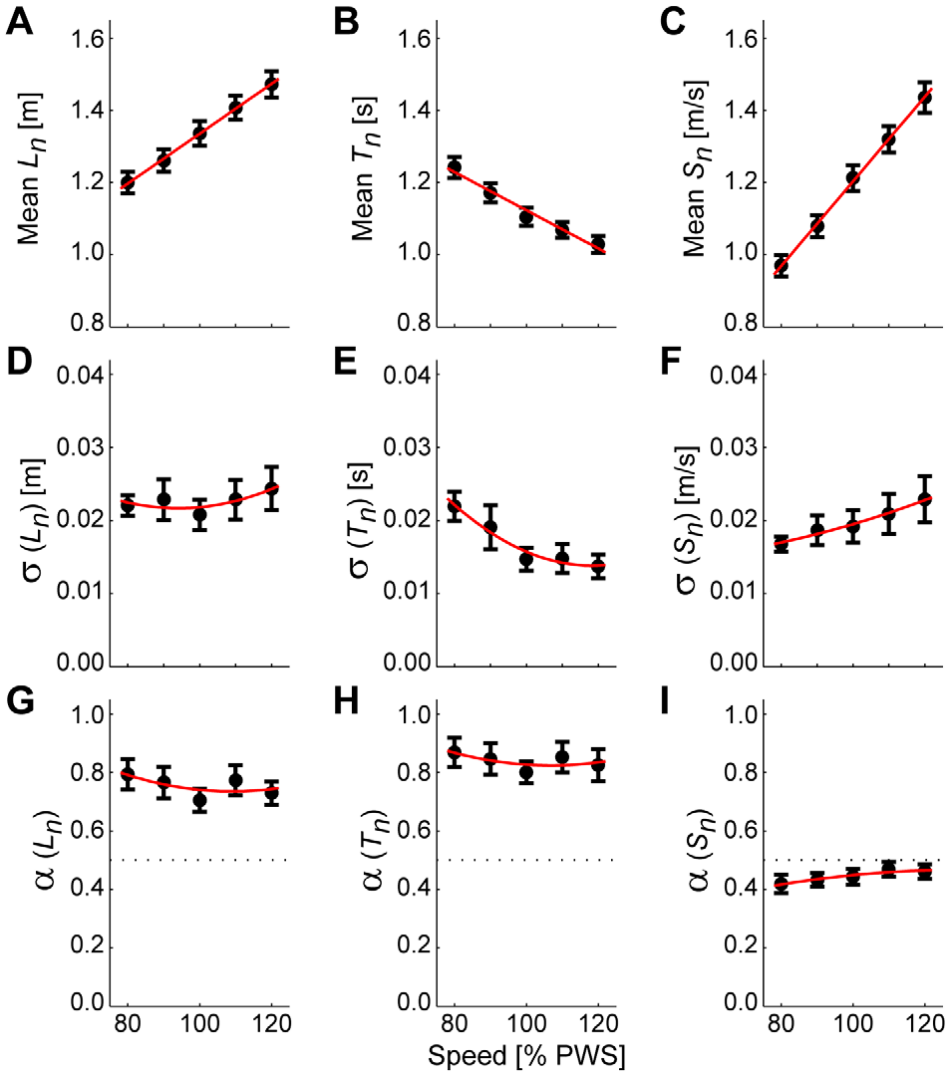
Primary gait parameters. Means (**A**, **B**, **C**), standard deviations (σ: **D**, **E**, **F**), and DFA exponents (*α*: **G**, **H**, **I**), for stride length (*L_n_*), stride time (*T_n_*), and stride speed (*S_n_*) as a function of walking speed from 80% to 120% of preferred walking speed (PWS). Error bars indicate between-subject ±95% confidence intervals at each speed. At faster walking speeds, subjects adopted longer stride lengths (**A**) and faster stride times (**B**). The variability in stride length (**D**) remained similar across speeds, while the variability in stride times (**E**) decreased at faster walking speeds. Consequently, the variability in the stride speeds (**F**) increased slightly at faster walking speeds. Subjects exhibited significant stride-to-stride statistical persistence (i.e., *α*>>½) in both stride lengths (**G**) and stride times (**H**), suggesting that deviations in these measures were not immediately corrected on consecutive strides. Conversely, subjects consistently exhibited slight *anti*-persistence (i.e., *α*<½) in stride speeds (**I**), suggesting that this measure of walking performance was under tighter control. Note: Linear trend lines in (**A**)–(**C**) and quadratic trend lines in (**D**)–(**I**) are shown only to provide a visual reference.

As expected, when subjects walked faster, they increased stride lengths (Fig. 3A), decreased stride times (Fig. 3B), and increased stride speeds (Fig. 3C). Stride length variability (Fig. 3D) increased slightly at speeds faster and slower than PWS, while stride time variability (Fig. 3E) increased at slower walking speeds, and stride speed variability (Fig. 3F) increased at faster walking speeds. However, standard deviations only quantify the average magnitude of differences across all strides, regardless of temporal order. They yield no information about how each stride affects subsequent strides.

Therefore, to quantify temporal correlations across consecutive strides, we computed scaling exponents, *α*, using Detrended Fluctuation Analysis (DFA) [22], [49], [51], [52] (see Methods). *α*>½ indicates statistical *persistence*: deviations in one direction are more likely to be followed by deviations in the same direction. *α*<½ implies *anti*-persistence: deviations in one direction are more likely to be followed by deviations in the opposite direction. *α* = ½ indicates uncorrelated noise: all deviations are equally likely to be followed by deviations in either direction. In the context of control, statistical persistence (*α*>½) is interpreted as indicating variables that are *not* tightly regulated. Conversely, variables that are tightly regulated are expected to exhibit either uncorrelated or anti-persistent fluctuations (*α*≤∼½).

Consistent with previous results [22], [50], [54], *T_n_* and *L_n_* time series (Figs. 3G, 3H) both exhibited significant statistical persistence (*α*>½). Conversely, *S_n_* time series (Fig. 3I) exhibited consistent and statistically significant *anti*-persistence (∼0.4<*α*<0.5). Thus, at all walking speeds, deviations in both *T_n_* and *L_n_* were allowed to persist, while deviations in *S_n_* were rapidly reversed on subsequent strides. This provides indirect evidence that subjects did not regulate *T_n_* or *L_n_* independently, but instead adjusted both *T_n_* and *L_n_* in a coordinated manner to maintain walking speed.

**Figure 4:**
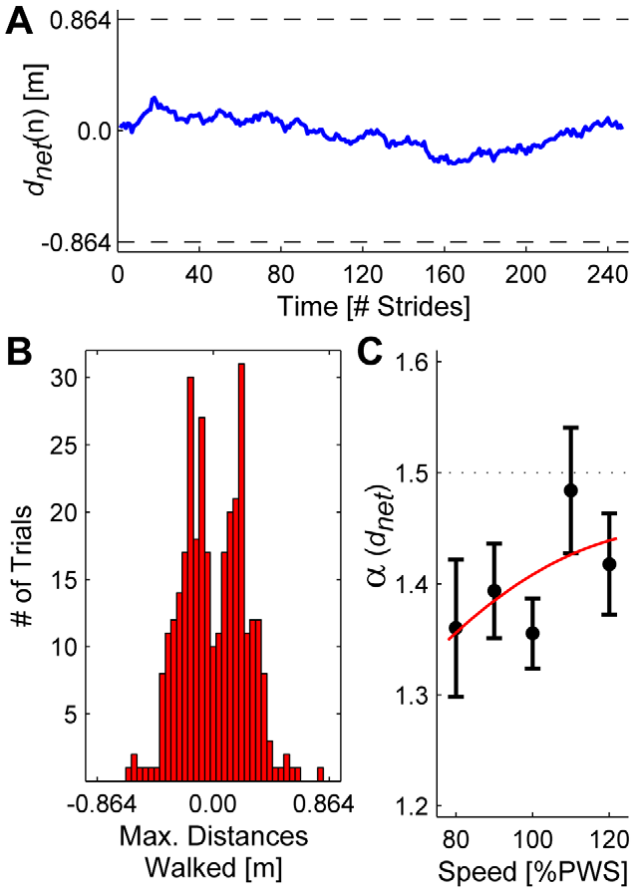
Absolute distances walked on the treadmill. (**A**) Net cumulative distance, *d_net_*(*n*), walked (i.e., absolute position, Eq. 6) on the treadmill over time for a typical trial for a typical human subject. Dashed horizontal lines at ±0.864 m indicate the front and back limits of the treadmill belt. All subjects exhibited substantial deviations in absolute position that were sustained across multiple strides, consistent with previous findings [23], [24], [55], [56]. (**B**) Histogram of maximum rearward (−) and forward (+) distances walked by each subject during each trial at all 5 speeds (166 total trials). Histograms for each individual speed looked similar. Note that most subjects did not get close to reaching the treadmill belt limits (±0.864 m). (**C**) These stride-to-stride shifts in absolute treadmill position exhibited very strong statistical persistence,approaching that of Brownian motion (i.e., integrated white noise: *α* = 1.5), particularly at the faster walking speeds. Thus, these deviations in absolute position were *not* tightly controlled. Note: the vertical scale here is quite different from Fig. 3G–I. The quadratic trend line is shown only to provide a visual reference.

As expected [23], [24], [55], [56], subjects did “drift” forward and backward (Eq. 1) over time along the treadmill belt (Fig. 4A). Most of these drifting movements remained contained within approximately the middle one third of the treadmill belt (Fig. 4B). This suggested that subjects adopted a more “conservative” walking strategy than actually *required* by the inequality constraint of Eq. (1). However, these movements also exhibited a *high* degree of statistical persistence (∼1.25<*α*<∼1.55) at all walking speeds (Fig. 4C). Thus, deviations in absolute position along the treadmill belt were allowed to persist even more so than deviations in either *T_n_* or *L_n_*. Thus, absolute position itself was *not* a tightly controlled variable for this task.

### GEM-Based Decomposition of Gait Variability

**Figure 5:**
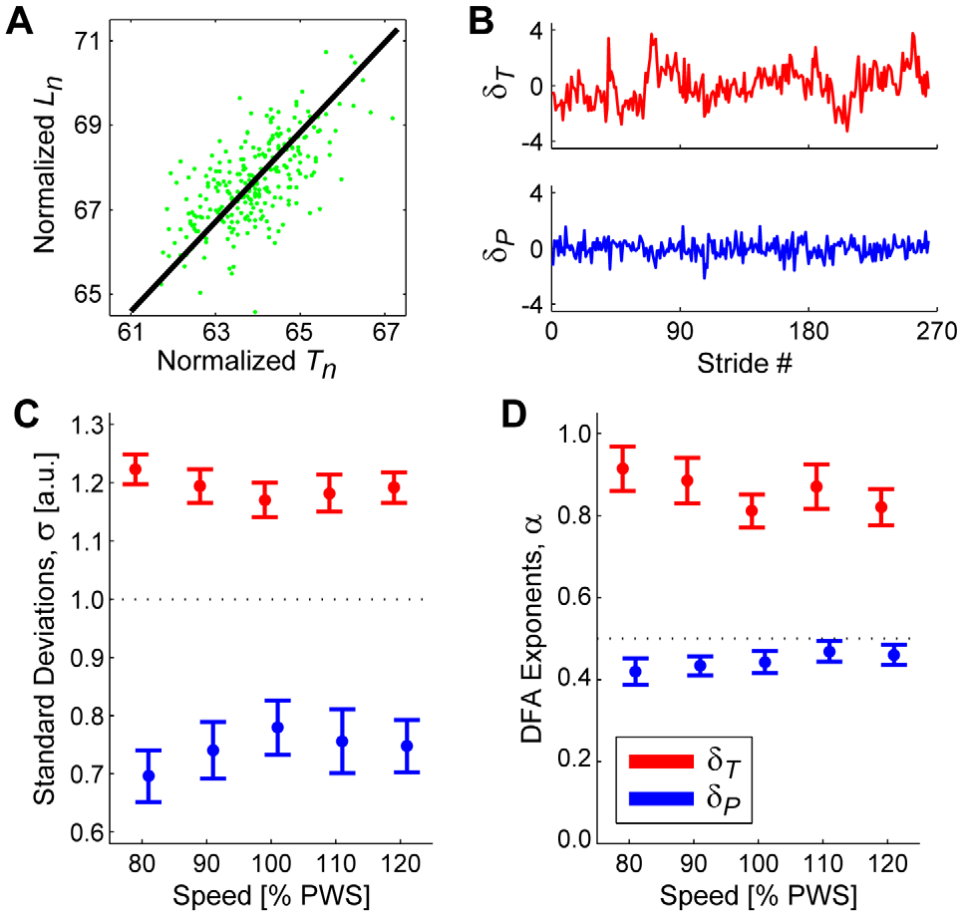
GEM decomposition results. (**A**) Example GEM data from a typical subject. Individual dots represent individual strides. The diagonal line represents the GEM (see Fig. 2). (**B**) Time series of *δ_T_* and *δ_P_* deviations for the data set shown in (**A**). Qualitatively, the *δ_T_* deviations exhibit larger amplitudes and also appear to show greater statistical persistence than the *δ_P_* deviations. (**C**) Standard deviations for all *δ_T_* and *δ_P_* time series at all 5 walking speeds. Error bars represent between-subject ±95% confidence intervals. Subjects exhibited significantly greater variability along the GEM (*δ_T_*) than perpendicular to the GEM (*δ_P_*): F_(1,16)_ = 139.93; p = 2.53×10^−9^. (**D**) DFA *α* exponents for all *δ_T_* and *δ_P_* time series at all 5 walking speeds. Error bars represent between-subject ±95% confidence intervals. Subjects exhibited significantly greater statistical persistence along the GEM (*δ_T_*) than perpendicular to the GEM (*δ_P_*): F_(1,16)_ = 368.21; p = 1.81×10^−12^. Additionally, all subjects exhibited significant anti-persistence (95% confidence interval upper bounds all <½) for the goal-relevant *δ_P_* deviations at all 5 walking speeds.

Plots of *L_n_* versus *T_n_* (e.g., Fig. 5A) exhibited distributions elongated along the GEM. As hypothesized, subjects exhibited far greater variability along the GEM than perpendicular to it (F_(1,16)_ = 139.93; p = 2.53×10^−9^; Fig. 5C). This contrasts with what would be expected if the distributions of [*T_n_*, *L_n_*] points were solely a reflection of average metabolic costs, given the nearly circular energy contours seen in Fig. 1. Additionally, the *δ_T_* time series all exhibited standard deviations >>1, while the *δ_P_* time series all exhibited standard deviations <<1 (Fig. 5C). Thus, subjects consistently exhibited much greater *δ_T_* variability and much less *δ_P_* variability than they did for either normalized (i.e., standard deviation = 1) *T_n_* or *L_n_* time series.

The *δ_T_* and *δ_P_* time series exhibited temporal correlation structures qualitatively very different from each other (Fig. 5B). As hypothesized, subjects exhibited far greater statistical persistence for *δ_T_* than for *δ_P_* (F_(1,16)_ = 368.21; p = 1.81×10^−12^; Fig. 5D). Additionally, all subjects exhibited significant statistical *anti*-persistence (i.e., 95% CI upper bounds for *α*<½) for the goal-relevant *δ_P_* deviations at all five walking speeds. Thus, subjects rapidly corrected *δ_P_* deviations from each stride to the next, while allowing *δ_T_* deviations to persist across multiple strides, independent of the magnitudes of these fluctuations.

### Surrogate Analyses – Plausible Alternative Strategies

One obvious question is whether these observed dynamics represented the *only* viable strategy subjects could have used. Rejecting this possibility requires only that we identify at least *one* alternative strategy that still satisfied the fundamental task requirements (Eq. 1), but was completely “ignorant” of the proposed GEM defined by Eq. 2. Here, we present *three* such alternatives using “surrogate” data [60], [61] that each represent the output of a particular type of *data-based model* of the observed stride-to-stride dynamics. Each surrogate model directly tested a specific null hypothesis that subjects could have successfully completed the treadmill walking task (i.e., satisfied Eq. 1) using a strategy that made absolutely *no* reference to the GEM.

**Figure 6:**
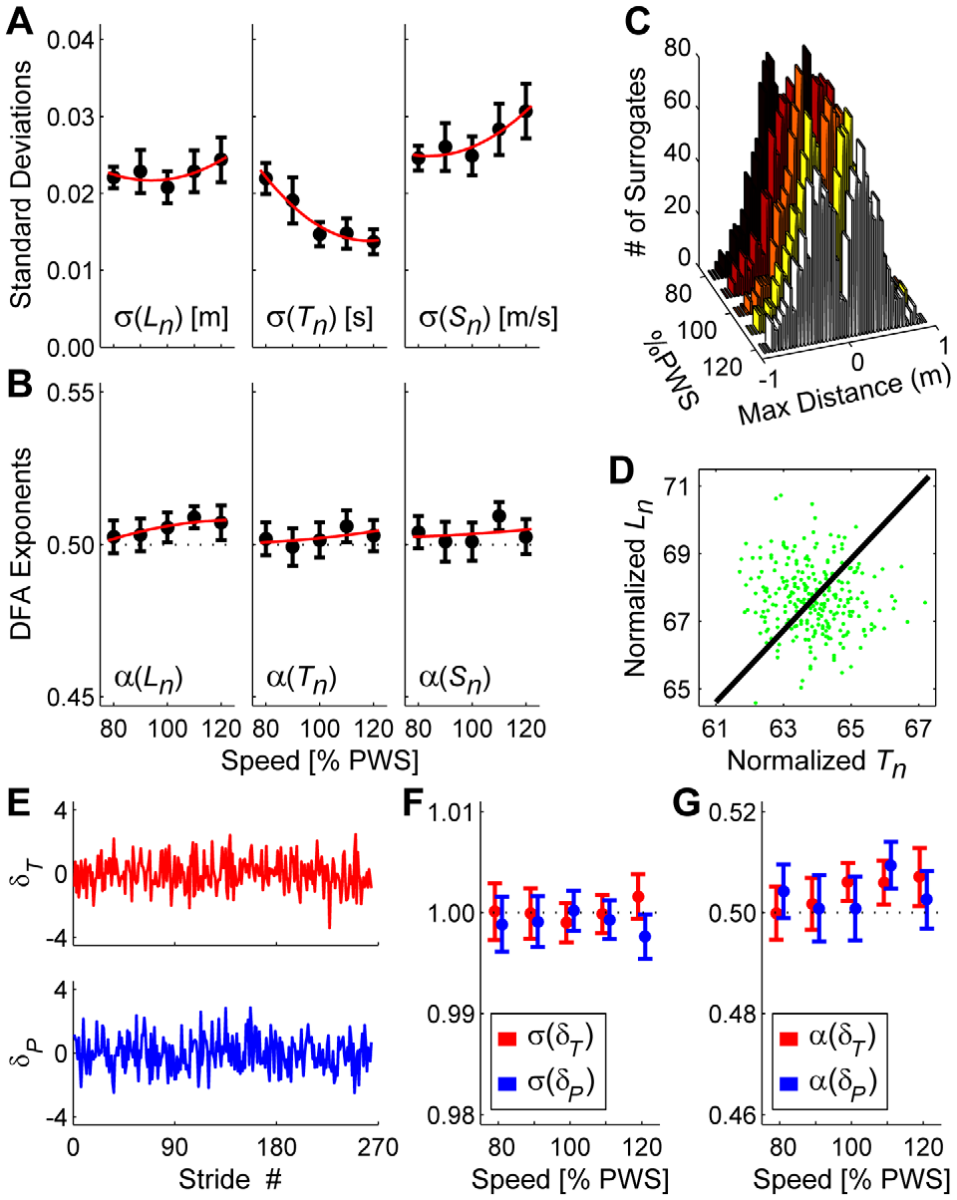
Independently randomly shuffled surrogate walking. All error bars represent between-subject ±95% confidence intervals. By definition, these surrogates exhibited the same mean stride parameters (not shown) as the original walking data (Fig. 3**A**–**C**). (**A**) These surrogates exhibited the same *L_n_* and *T_n_* variability as the original data (Fig. 3**D**–**E**). However, *S_n_* variability increased slightly (compare to Fig. 3**F**). (**B**) Unlike the experimental trials (Fig. 3**G**–**I**), these surrogates exhibited no strong temporal correlations (all *α*≈½) for any of the basic stride parameters (Note, the vertical scale is *very* different from Fig. 3**G**–**I**). (**C**) Histograms of maximum forward and backward distances walked by all 20 surrogates for each trial. By construction, no surrogate walked beyond either the front or back edges of the treadmill belt (i.e., ±0.864 m). (**D**) A typical surrogate for the trial shown in Fig. 5**A**. The GEM (diagonal line) remains the same. However, the distribution of strides around the GEM is now approximately isotropic. (**E**) Time series of *δ_T_* and *δ_P_* deviations for the surrogate trial shown in (**D**). Neither time series exhibited obvious persistence. (**F**) Variability (σ) for *δ_T_* and *δ_P_* deviations from the GEM was not significantly different (F_(1,16)_ = 2.614; p = 0.125) (Compare to Fig. 5**C** and note the different vertical scales). (**G**) There were no strong temporal correlations (*α*≈½) for either *δ_T_* or *δ_P_* deviations and *α*'s for both directions were not different from each other (F_(1,16)_ = 0.413; p = 0.529) (Compare to Fig. 5**D** and note the different vertical scales).

The first alternative strategy was to choose a reference point, [*T* ^*^, *L* ^*^] (e.g., Fig. 1), on the GEM and maintain sufficiently small variance about this point to satisfy Eq. (1). Here, “control” would consist entirely of suppressing variability in both *L_n_* and *T_n_* caused by neuro-motor noise. This controller would therefore be completely ignorant of the GEM. We implemented this hypothetical controller by generating 20 randomly shuffled surrogates [22], [60], [61] for each experimental trial. This procedure maintained the exact same means and variances of the original *L_n_* and *T_n_* time series (Fig. 6A). However, all effects of temporal order were eliminated, yielding statistically uncorrelated time series (*α*≈½; Fig. 6B). By construction, all surrogates were constrained to not “walk off” the front or back end of the treadmill (Fig. 6C), thus satisfying Eq. 1.

These surrogates exhibited approximately isotropic distributions (i.e., no obvious directionality) about [*T* ^*^, *L* ^*^] within the [*T_n_*, *L_n_*] plane (Fig. 6D). Likewise, *δ_P_* and *δ_T_* time series were qualitatively very similar to each other (Fig. 6E). Standard deviations for *δ_P_* and *δ_T_* were both≈1 and not significantly different (F_(1,16)_ = 2.614; p = 0.125; Fig. 6F). DFA *α* exponents for *δ_P_* and *δ_T_* were both≈½ and also not significantly different (F_(1,16)_ = 0.413; p = 0.529; Fig. 6G). Most importantly, these surrogates exhibited statistical and dynamical properties drastically different from the experimental data (Fig. 5). Thus, the null hypothesis that subjects used this alternative “GEM ignorant” strategy to accomplish the treadmill walking task was rejected.

Fig. 6 demonstrates unequivocally that the strategy subjects used (Fig. 5) was not the only successful strategy they could have adopted. They could have adopted a control policy that equally achieved the task requirement defined by Eq. 1 without using the GEM-based control strategy defined by Eq. 2. We also used surrogate data techniques to test two additional model hypotheses of how subjects might have controlled their stride-to-stride dynamics. We tested a second alternative strategy that also regulated *T_n_* and *L_n_* independently of the GEM, but in a way that retained the statistical persistence observed in humans (Fig. 3G,H) [22], [53], [54]. We then tested a third possibility that the covariation observed in [*T_n_*, *L_n_*] (Figs. 5A,C) was *not* due to stride-to-stride “control,” but to simple biomechanics [42]: i.e., taking longer (or shorter) *L_n_* naturally required longer (or shorter) *T_n_*. Subjects did *not* adopt either of these two viable alternative control strategies. Full details and results of these analyses are presented in Supplementary Text S2.

### Stochastic Optimal Control Models

To obtain more definitive conclusions about the underlying control policies used, we first hypothesized that subjects controlled their movements based on the minimum intervention principle (MIP) [27], [28], [41], [42]. We created a model “walker” (see Methods), where a two-dimensional state variable, **x** *_n_* = [*T_n_*, *L_n_*]*^T^*, defined each stride. We implemented a stochastic optimal control policy that directly corrected *δ_P_* deviations at each stride, but ignored *δ_T_* deviations.

**Figure 7 pcbi-1000856-g007:**
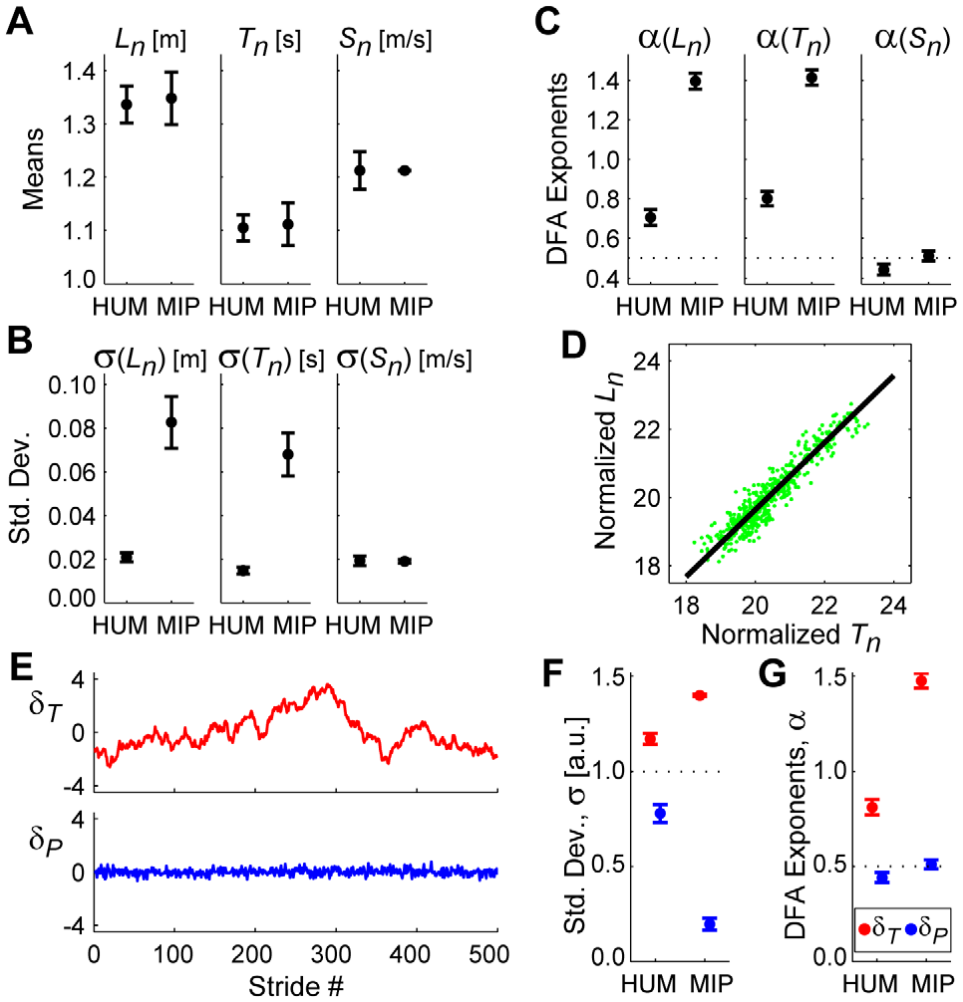
Stochastically optimal minimum intervention principle (MIP) model for step regulation. All error bars represent between-subject ±95% confidence intervals. In (**A**)–(**C**) and (**F**)–(**G**), HUM data are the experimental data from Fig. 3 for 100% PWS. (**A**) Mean stride lengths (*L_n_*), times (*T_n_*) and speeds (*S_n_*) for humans (HUM) and for the MIP model (MIP). (**B**) Within-subject standard deviations for *L_n_*, *T_n_*, and *S_n_*. (**C**) DFA exponents (*α*) for *L_n_*, *T_n_*, and *S_n_*. (**D**) A typical trial for the MIP model. The diagonal line represents the GEM. As expected, the distribution of strides is very tightly compressed along the GEM. (**E**) Time series of *δ_T_* and *δ_P_* deviations for the trial shown in (**D**). Note the substantial statistical persistence exhibited by the *δ_T_* time series. (**F**) Variability (σ) for the MIP model data was significantly greater for *δ_T_* deviations than for *δ_P_* deviations (F_(1,39)_ = 6,076.51; p = 1.53×10^−43^). The MIP model exhibited much greater *δ_T_* variability and much less *δ_P_* variability than did human subjects (HUM). (**G**) DFA exponents (*α*) for the MIP model were significantly larger for *δ_T_* deviations than for *δ_P_* deviations (F_(1,39)_ = 1,969.18; p = 2.40×10^−34^). DFA exponents (*α*) for *δ_T_* deviations were ∼1.5, reflecting Brownian motion (i.e., statistical diffusion) along the GEM. Conversely, *α* exponents for the *δ_P_* deviations were ∼½, reflecting nearly uncorrelated fluctuations. These goal-relevant *δ_P_* deviations did *not* exhibit the anti-persistent behavior seen in the experimental data (Fig. 5**D**).

By construction, this MIP model walked with nearly the same average stride parameters (Fig. 7A) and stride speed (*S_n_*) standard deviations (Fig. 7B) as humans. However, the MIP model exhibited substantially greater variability in both *L_n_* and *T_n_* (Fig. 7B). The MIP model also exhibited much greater statistical persistence for *L_n_* and *T_n_* than humans, while *S_n_* was statistically uncorrelated (Fig. 7C). Data points were aligned very closely to the GEM (Fig. 7D). The *δ_T_* time series exhibited both much greater variability (F_(1,39)_ = 6,076.51; p = 1.53×10^−43^; Fig. 7E,F) and more persistent fluctuations (F_(1,39)_ = 1,969.18; p = 2.40×10^−34^; Fig. 7E,G) than did *δ_P_*. Because *no* control effort was applied along the GEM, consecutive strides exhibited approximately random walk behavior, or Brownian motion, (i.e., *α* ≈1.5) in *δ_T_*. Thus, our hypothesis that subjects adopted this stochastically optimal MIP control [27], [28] was rejected.

**Figure 8 pcbi-1000856-g008:**
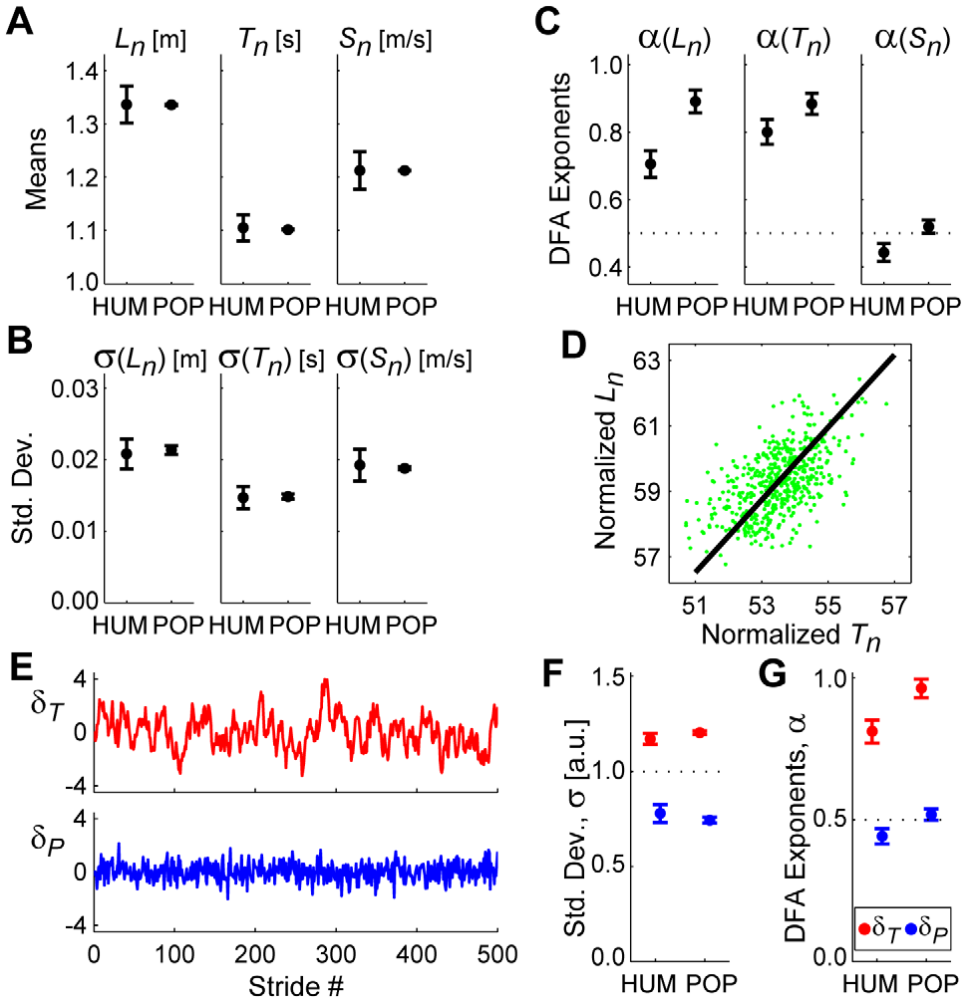
Stochastically optimal MIP-based model with “preferred operating point” (POP) for step regulation. All error bars represent between-subject ±95% confidence intervals. In (**A**)–(**C**) and (**F**)–(**G**), HUM data are the experimental data from Fig. 3 for 100% PWS. (**A**) Mean stride lengths (*L_n_*), times (*T_n_*) and speeds (*S_n_*) for humans (HUM) and for the POP model. (**B**) Within-subject standard deviations for *L_n_*, *T_n_*, and *S_n_*. (**C**) DFA exponents (*α*) for *L_n_*, *T_n_*, and *S_n_*. (**D**) A typical POP model trial. The diagonal line represents the GEM. As expected, the distribution of strides is not nearly as compressed along the GEM as for the MIP model (Fig. 7**D**). (**E**) Time series of *δ_T_* and *δ_P_* deviations for the trial shown in (**D**). The *δ_T_* time series appears to exhibit persistence. The *δ_P_* time series does not. (**F**) Variability (σ) for the POP model was still greater for *δ_T_* deviations than for *δ_P_* deviations (F_(1,39)_ = 2,916.30; p = 1.55×10^−37^). However, the variance ratio, σ(*δ_T_*)/σ(*δ_P_*), was much closer that of humans. (**G**) DFA exponents (*α*) for the POP model were significantly larger for *δ_T_* deviations than for *δ_P_* deviations (F_(1,39)_ = 597.27; p = 7.61×10^−25^). For *δ_T_* deviations, these *α* were still >1.0, reflecting substantial statistical persistence. Likewise, the *α* for *δ_P_* deviations were still ∼½, reflecting uncorrelated fluctuations. The *δ_P_* deviations still did *not* exhibit the anti-persistent behavior seen experimentally (Fig. 5**D**).

However, the MIP model did not incorporate any additional physiological and/or biomechanical constraints. Because human legs have finite length, they cannot take extremely long steps easily. Because they have inertia, they cannot easily move extremely fast. Likewise, the MIP model incorporated no capacity to minimize energy cost [5]–[12]. Each of these factors would act to constrain the choices of *L_n_* and *T_n_* to a smaller range along the GEM. We therefore hypothesized that subjects adopted a different MIP-based control policy that also used a “preferred operating point” (POP) on the GEM, where this POP, [*T* ^*^, *L* ^*^], was assumed to be equal to the mean stride time and stride length (Fig. 8).

By construction, this POP model also walked with nearly the same average stride parameters (Fig. 8A) and variability (Fig. 8B) as humans. Likewise, this model exhibited statistical persistence (*α*>½) for both *L_n_* and *T_n_* that, while still greater, were much closer to those of humans (Fig. 8C). This model did not, however, capture the anti-persistence (*α*<½) exhibited by humans for *S_n_* (Fig. 8C). The POP model exhibited greater relative *δ_P_* variability than did the MIP model (Fig. 8D,E), very similar to humans (Fig. 8F). The magnitudes of the *δ_T_* fluctuations were much greater than those of the *δ_P_* fluctuations (F_(1,39)_ = 2,916.30; p = 1.55×10^−37^; Fig. 8F). This model also exhibited larger DFA *α* exponents for *δ_T_* fluctuations than for *δ_P_* fluctuations (F_(1,39)_ = 597.27; p = 7.61×10^−25^; Fig. 8G). As expected, *α* exponents for *δ_T_* were greatly reduced compared to the MIP model. However, this model still failed to replicate the *anti*-persistent (*α*<½) *δ_P_* fluctuations exhibited by humans (Fig. 8G). Thus, our hypothesis that subjects adopted this modified control policy was partly supported, but ultimately rejected.

**Figure 9:**
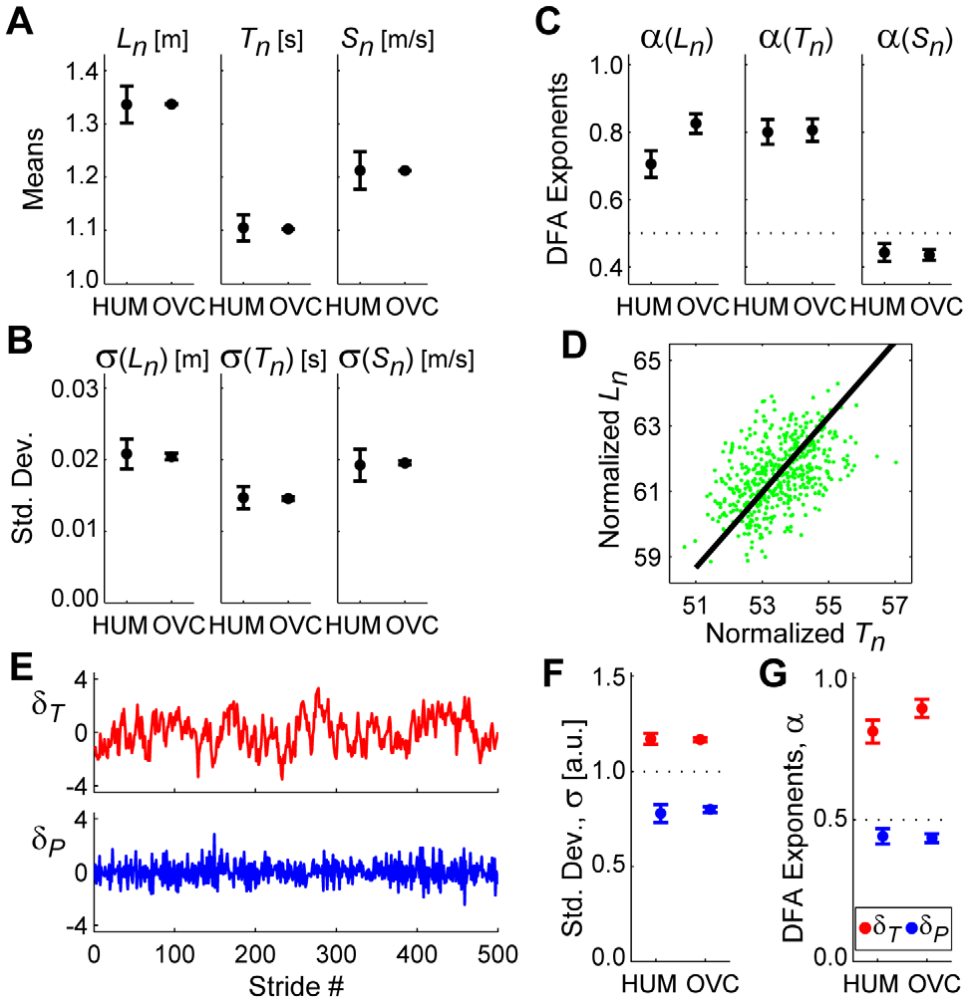
Sub-optimal MIP-based model with “over-correcting” (OVC) controller for step regulation. All error bars represent between-subject ±95% confidence intervals. In (**A**)–(**C**) and (**F**)–(**G**), HUM data are the experimental data from Fig. 5 for 100% PWS. (**A**) Mean stride lengths (*L_n_*), times (*T_n_*) and speeds (*S_n_*) for humans (HUM) and OVC model (OVC). (**B**) Within-subject standard deviations for *L_n_*, *T_n_*, and *S_n_*. (**C**) DFA exponents (*α*) for *L_n_*, *T_n_*, and *S_n_*. (**D**) A typical OVC model trial. The diagonal line represents the GEM. The distribution of strides with respect to the GEM appears similar to the POP model (Fig. 8**D**) and to humans (Fig. 5**A**). (**E**) Time series of *δ_T_* and *δ_P_* deviations for the trial shown in (**D**). The *δ_P_* time series now appears to exhibit slightly more rapid fluctuations than did the POP model (Fig. 8**E**). (**F**) Variability (σ) for the OVC model was much greater for *δ_T_* deviations than for *δ_P_* deviations (F_(1,39)_ = 1,736.81; p = 2.49×10^−33^). The variance *ratio*, σ(*δ_T_*)/σ(*δ_P_*), was again very similar to humans. (**G**) DFA exponents (*α*) for the OVC model were significantly larger for *δ_T_* deviations than for *δ_P_* deviations (F_(1,39)_ = 713.02; p = 3.15×10^−26^). Deviations along the GEM (*δ_T_*) again exhibited statistical persistence. Conversely, the *δ_P_* deviations consistently exhibited *α*<½. Thus, these *δ_P_* deviations *did* exhibit the anti-persistent behavior seen experimentally (Fig. 5**D**).

The MIP and POP models both optimally corrected deviations away from the GEM at the next stride. Thus, the *δ_P_* fluctuations in each case (Figs. 7G, 8G) reflected nearly uncorrelated white noise (*α*≈½). Conversely, humans consistently exhibited statistical *anti*-persistence (*α*<½) in their *δ_P_* fluctuations (Fig. 5D). This suggests that humans corrected these *δ_P_* deviations more than would be expected from a single stride optimal control policy. To test this hypothesis, we implemented an “OVC” controller that slightly *over*-corrected *δ_P_* deviations at each successive stride (Fig. 9).

By construction, this OVC model walked with nearly the same average stride parameters (Fig. 9A), stride variability (Fig. 9B), and statistical persistence for both *T_n_* and *L_n_* (Fig. 9C) as humans. Unlike the MIP and POP models, this OVC model *did* capture the anti-persistence (*α*<½) exhibited by humans for *S_n_* (Fig. 9C). The OVC model yielded GEM decomposition results qualitatively (Figs. 9D,E) and quantitatively (Figs. 9F,G) consistent with humans. Most importantly, this model now exhibited the anti-persistent *δ_P_* fluctuations (Fig. 9G) observed in humans (Fig. 5D). Thus, our hypothesis that subjects adopted a control policy that slightly *over*-corrected deviations away from the GEM was supported.

## Discussion

This study set out to determine how humans regulate stride-to-stride variations in treadmill walking. We specifically sought to determine if the nervous system always overcomes all variability as a fundamental performance limitation [16], [26], [29], [32], or if it instead exploits redundancy to *selectively* regulate the effects of variability and enhance task performance [25], [27], [28]. We demonstrate that formulating mathematical hypotheses on specific strategies (e.g., Eq. 2) used to achieve task requirements (e.g., Eq. 1) can reconcile issues of optimality, redundancy, and stochasticity in human walking. Our results reveal a new governing principle for regulating stride-to-stride fluctuations in human walking that acts *independently* of, but in parallel with, the principle of minimizing energy cost [5]–[12].

We hypothesized that humans walking on a treadmill would adopt a specific strategy [25], [57] to maintain constant speed at each consecutive stride (Eq. 2), something *not* absolutely required to complete this task. This yielded a decomposition of stride-to-stride variations into new gait variables (*δ_P_* and *δ_T_*) (Fig. 2). Human subjects adjusted their steps specifically to achieve this hypothesized strategy (Fig. 5). Moreover, they did so across a range of walking speeds, demonstrating that this strategy is robust to alterations in task requirements. Subjects did *not* use perfectly viable alternative strategies, including three that completely ignored the GEM (Figs. 6 and Supplementary Text S2), and two based on optimal control models (Figs. 7–8). Instead, stride-to-stride dynamics were directly consistent with a control strategy that first seeks to minimize goal-relevant *δ_P_* errors (Fig. 7) [25], [27], but then also weakly limits *δ_T_* variations (Fig. 8) and slightly *over*-corrects *δ_P_* deviations (Fig. 9). These results confirm that the neuromotor control of treadmill walking is organized around the hypothesized goal function (Eq. 2).

Beyond the five alternative control strategies clearly rejected by our results (Figs. 6–8 and Supplementary Text S2), other plausible alternatives were considered. One seemingly reasonable strategy might be to try to stay at a fixed location on the treadmill. Such absolute position control would necessitate regulating *d_net_*(*n*) (see Methods, Eq. 4), in contrast with the controllers derived here that regulate stride *speed*, (Eq. 2). However, the statistical persistence in the experimental *d_net_*(*n*) data (Fig. 4A,C) strongly suggests that people do not regulate their walking this way. Our stochastic optimal control models demonstrate that the level of control strongly determines the statistical persistence of a time series. For both the MIP and POP models (Figs. 7–8), stochastic optimal control with respect to the hypothesized GEM (Eq. 2) yielded *δ_P_* fluctuations with *α*(*δ_P_*)≈½ (Figs. 7G & 8G). *Increasing* the control gains above unity for the OVC model (so the model *over*-corrected errors in *δ_P_*) yielded *α*(*δ_P_*)<½ (Fig. 9G). Likewise, *decreasing* these control gains (so the model *under*-corrected errors in *δ_P_*) would yield *α*(*δ_P_*)>½. This phenomenon was also observed *along* the GEM. The POP and OVC models that applied *weak* control along the GEM yielded ½<*α*(*δ_T_*)<1 (Figs. 8G & 9G). The MIP model that applied *no* control along the GEM yielded *α*(*δ_T_*)≈1½ (Fig. 7G), as predicted. A value of *α* = 1½ corresponds to Brownian motion, where each deviation is simply a random change from the previous value. Thus, a position controller that minimized *d_net_*(*n*) in a stochastically optimal way would similarly yield *α*(*d_net_*)≈½. This was clearly *not* observed in our experiments, where we instead found *α*(*d_net_*)≈1½ (Fig. 4C). Thus, the possibility of absolute position control was also rejected in favor of speed control.

Minimizing energy cost has been the primary explanation for how humans and animals regulate walking [5]–[12]. This criterion predicts the presence of a single optimal operating point, [*T_Opt_*, *L_Opt_*], in the [*T_n_*, *L_n_*] plane [7], [9], [10]. Deviations away from [*T_Opt_*, *L_Opt_*], induced for example by neuromuscular noise [31], [45]–[47], would increase energy cost approximately equally for equivalent relative changes in all directions (Fig. 1). If variability were merely a limiting constraint the central nervous system must overcome [16], [26], [29], [32], the distributions of the variations around [*T_Opt_*, *L_Opt_*] should, on average, approximate the shape of the contours shown in Fig. 1 to minimize deviations from [*T_Opt_*, *L_Opt_*]. We did *not* observe that here. Instead, all [*T_n_*, *L_n_*] data were strongly oriented along the GEM (Fig. 3A,C). Indeed, the failure of the surrogates (Fig. 6) to capture the experimentally observed gait dynamics clearly refutes the idea that humans *only* try to minimize variations in [*T_n_*, *L_n_*] about a single operating point. Instead, while subjects rapidly corrected *δ_P_* deviations, they allowed *δ_T_* deviations to persist (Fig. 5B,D), even though these deviations would increase energy cost.

Our findings, however, remain compatible with the idea that humans also try to minimize energy cost while walking. The failure of the MIP model (Fig. 7) to capture the experimentally observed gait dynamics demonstrates that humans do not *only* minimize deviations away from the GEM. The POP model (Fig. 8), is precisely compatible with adding the secondary goal of minimizing energy cost. For the average walking speed modeled (*v* = 1.21.m/s), we computed a POP of [*T* ^*^, *L* ^*^] = [1.105 s, 1.337 m]. Mechanical walking models of Minetti [9] and Kuo [10] predict similar energetically optimal POPs of [*T_Opt_*, *l_Opt_*] = [1.029 s, 1.247 m] and [*T_Opt_*, *L_Opt_*] = [1.013 s, 1.228 m], respectively, for this speed. Simplifications in both models account for their slightly under-estimating the preferred [*T* ^*^, *L* ^*^] of actual humans [9].

Humans also consistently *over*-corrected *δ_P_* deviations (Fig. 5D). Our OVC model (Fig. 9) provides one possible explanation: that humans use sub-optimal control to correct stride-to-stride deviations. In the model, anti-persistence in *δ_P_* implies sub-optimal and vice-versa. More importantly, data analysis methods currently used to substantiate UCM [39], [40] and MIP [18], [27], [28], [42] predictions would not have captured this because they only consider *variability* in the data. However, taken alone, our variability results are entirely compatible with either the optimal POP (Fig. 8F) or sub-optimal OVC (Fig. 9F) controllers. Only the DFA analyses (Figs. 8G, 9G) allow us to distinguish these two models, by offering an additional measure of stride-to-stride *dynamics* [57], [62] that is independent of variability [22], [49], [51]. Perhaps most explicitly, the paired surrogates (see Supplementary Text S2) exhibited very strong alignment of variance along the GEM, even though these surrogates, by definition, represented an explicitly GEM-*ignorant* control strategy. Thus, quantifying variance ratios alone (as done in experimental applications of UCM and MIP) can very easily lead to incorrect conclusions about control (see also [42]). Our results demonstrate that it is critical to quantify both variability *and* temporal dynamics [57], [62] to fully determine how repetitive movements are controlled.

The principal contribution of our work is thus to demonstrate that considerations other than minimizing energy cost help determine [*T_n_*, *L_n_*] at each stride. Subjects instead choose [*T_n_*, *L_n_*] based on a hierarchy of defined goals [25], with at least one short-term goal to maintain walking speed, and one long-term goal to reduce energy cost. Humans adopt GEM-aware control over short (stride-to-stride) time scales, while still minimizing energetic cost over longer (on average) time scales. They readily exploit this [*T_n_*, *L_n_*] redundancy during level treadmill walking, even though they do not have to (Fig. 6 and Supplementary Text S2). This ability to fully exploit the redundancy available could become critical when tasks become more demanding. In walking for example, rapidly and effectively adjusting successive steps could become critical when negotiating uneven terrain [63]. However, these adjustments need to be made at *each* step and not just on average. Thus, GEM-aware control exploits inherent task redundancy [25], [27], [28] to simultaneously achieve high task performance (low error) while allowing possibly beneficial motor variability.

The nervous system appears to estimate both motor errors and the sources of those errors to guide continued adaptation [30], [31], [33]. The neural structures involved in decision making may even deliberately insert noise into the process to enhance adaptation [64], [65]. Exposing humans to tasks that share similar structural characteristics but vary randomly may even help facilitate the ability to generalize to novel tasks [33]. Similar capacities were recently demonstrated even in highly-learned (i.e., “crystallized”) adult bird song [66], where residual variability in this skill represented “meaningful motor exploration” to enhance continued learning and performance optimization [31], [66], [67]. Our findings suggest that similar purposeful motor exploration occurs in the highly-learned task of human walking.

It has been widely argued that statistically persistent fluctuations are a critical marker of “healthy” physiological function [51], [52] and that uncorrelated or anti-persistent fluctuations are a sign of disease or pathology [51]–[53]. The present results strongly refute this interpretation. The subjects tested here clearly cannot be simultaneously both “healthy” (according to *α*(*δ_T_*)) and “unhealthy” (according to *α*(*δ_P_*)) (Fig. 5D). Instead, our findings argue for interpreting these DFA exponents specifically within the context of the control processes involved (Figs. 7–9). This interpretation is fully consistent with the fact that many random processes can yield time series with a wide range of *α* values [68]. In previous work, this was directly supported by a simple mechanical model of walking with minimal feedback control that still exhibited a wide range of statistically persistent and anti-persistent walking behaviors [62].

One question is whether the theoretical framework developed here will generalize to other contexts. During unconstrained overground walking [50], humans exhibited strong statistical persistence for *T_n_* and *L_n_* similar to Fig. 5G–H. However, unlike Fig. 5I, they also exhibited strong persistence for *S_n_* [50]. When those subjects walked in time with a metronome, *L_n_* and *S_n_* remained strongly persistent [50], but *T_n_* became *anti*-persistent [50], [69], [70]. All three results (treadmill, overground, and metronome) are precisely compatible with the idea that humans adopt generalized “Minimum Intervention” [27] strategies to tightly regulate only those variables that are directly relevant to achieving some specified task goal [25]. On the treadmill, humans tightly regulate walking *speed* (Fig. 5). Remove the treadmill, and subjects no longer tightly regulate any one individual stride parameter [50]. Introduce a metronome, and subjects tightly regulate gait cycle *timing* (*T_n_*), but not *L_n_* or *S_n_* [50]. In all three contexts, factors *beyond* minimizing energy cost help determine how stride-to-stride movements are regulated. The critical first step is to identify the appropriate *goal function* for each task [25].

## Methods

### Ethics Statement

All participants provided written informed consent, as approved by the University of Texas Institutional Review Board.

### Subjects and Protocol

Seventeen young healthy adults (12M/5F, age 18–28, height 1.73±0.09 m, body mass 71.11±9.86 kg), participated. Subjects were screened to exclude anyone who reported any history of orthopedic problems, recent lower extremity injuries, any visible gait anomalies, or were taking medications that may have influenced their gait.

Subjects walked on a level motor-driven treadmill (Desmo S model, Woodway USA, Waukesha WI) while wearing comfortable walking shoes and a safety harness (Protecta International, Houston TX) that allowed natural arm swing [44]. First, preferred self-selected walking speed (PWS) was determined [23]. Subjects reported the limits of their PWS while the treadmill was slowly accelerated and then decelerated three times. These upper and lower limits were averaged to determine PWS [23]. Following a 2-minute rest, subjects completed two 5-minute walking trials at each of five speeds (80, 90, 100, 110 and 120% of PWS), presented in pseudo-random order [44]. Subjects rested at least 2 minutes between each trial to prevent fatigue. Subjects were instructed to look ahead and avoid extraneous movements while walking. Data from 1 trial from each of 4 subjects (i.e., 2.35% of all 170 trials collected) were discarded due to poor data quality. For the remaining 166 trials, an average of 272±25 total strides (range: 213–334) were analyzed.

### Data Collection and Processing

Five 14-mm retro-reflective markers were mounted to each shoe (heads of the 2^nd^ phalanx and 5^th^ metatarsal, dorsum of the foot, inferior to the fibula, and calcaneous). The movements of these markers were recorded using an 8-camera Vicon 612 motion capture system (Oxford Metrics, UK). All data were processed using MATLAB 7.04 (Mathworks, Natick MA). Brief gaps in the raw kinematic recordings were filled using rigid-body assumptions. Marker trajectories were low-pass filtered with a zero-lag Butterworth filter at a cutoff frequency of 10 Hz. A heel strike was defined as the point where the heel marker of the forward foot was at its most forward point during each gait cycle.

For the present analyses, the relevant walking dynamics were entirely captured by the impact Poincaré [58], [59] section defined by the [*T_n_*, *L_n_*] plane (Fig. 1). Thus, stride time (*T_n_*) for each stride, *n*, was calculated as the time from one heel contact to the next ipsilateral heel contact. Step length was defined as the anterior-posterior distance between the heel and the contralateral heel at each heel contact, when both feet were in contact with the treadmill belt. Stride length (*L_n_*) was calculated as the sum of the 2 consecutive step lengths composing each stride. Individual stride speeds (*S_n_*) were then calculated as 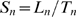. Average walking speed was computed as the average stride speed, 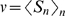, where 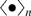 denotes the average over all *n* strides. Means, standard deviations, and DFA scaling exponents (*α*, see below) were computed across all strides for each *T_n_*, *L_n_*, and *S_n_* time series obtained from each walking trial (Fig. 3).

### GEM Decomposition

*T_n_* and *L_n_* were first normalized to unit variance (Fig. 1B) by dividing each time series by its own standard deviation (Fig. 3D–E). This provided an intuitive reference (σ = 1) for comparisons. We explored the effects of performing several different normalizations, but these did not change our results. In fact, it can be shown analytically that renormalizations of similar magnitude for both variables (as done here) have no discernable effect on our results. Using different normalizations of similar magnitudes for both variables would change the values of the axis labels, but would not change how the data were *distributed* in these plots. For example, dividing all stride lengths and times in Fig. 1B by 10 would change the axis labels, but the graph itself would still look identical. The GEM and 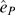 and 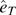 unit vectors also re-scale accordingly. If we used different normalizations (with similar magnitudes for both variables), the *values* of the standard deviations would change, but the *relative* differences in variability (e.g., Fig. 5C, etc.) would not. Since DFA *α* exponents are already unitless, these measures (e.g., Fig. 5D, etc.) retain their same *actual values* as well.

We defined a specific operating point on each GEM as 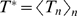 and 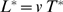, and defined new coordinates centered at this operating point, 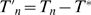 and 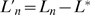. We then performed a linear coordinate transformation to define the deviations along the GEM, *δ_T_*, and perpendicular to the GEM, *δ_P_* (Fig. 1):
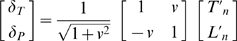
(3)Standard deviations and DFA scaling exponents (*α*, see Supplementary Text S4) were computed across all strides for each *δ_T_* and *δ_P_* time series obtained from each walking trial (Fig. 5).

### Surrogate Time Series and Analyses

Three types of surrogate time series [60], [61] were generated and analyzed. First, *randomly shuffled* surrogates (Fig. 6) were generated for each trial by independently shuffling each original *T_n_* and *L_n_* time series in random order. These surrogates retained the exact same mean, variance, and probability distribution of the original time series, while eliminating all effects of temporal order and any correlations between *T_n_* and *L_n_*. Randomly shuffled surrogates tested an alternative control model where subjects choose stride times and stride lengths that were independent of each other and the GEM, and temporally independent from each stride to the next.

Second, *phase-randomized* surrogates [43], [60], [61] were generated separately for the original *T_n_* and *L_n_* time series for each trial (see Supplementary Text S2). These surrogates tested an alternative control model where subjects choose stride times and stride lengths that were independent of each other and the GEM, but that remained temporally correlated across consecutive strides.

Third, for each trial *paired* randomly shuffled surrogates were generated simultaneously by randomly shuffling both *T_n_* and *L_n_* in exactly the same way (see Supplementary Text S2). These surrogates tested an alternative control model where stride times and stride lengths may have been coupled mechanically, but were still chosen independently of the GEM and independently from each stride to the next.

All surrogates were constrained so they did not “walk off” the treadmill (i.e., *all* surrogates satisfied Eq. 1). This was easily verified by computing the net cumulative distance (*d_net_*) each surrogate time series would have walked relative to the treadmill at each stride, *n*:
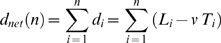
(4)where *d* = 0 represents the center of the treadmill belt. We then extracted the maximum forward [max(*d_net_*)], and backward [min(*d_net_*)] distances each surrogate walked during the entire trial (e.g., Fig. 6C). In this way, we confirmed that none of the surrogates walked off the treadmill (i.e., min(*d_net_*)≥−0.864 m and max(*d_net_*)≤+0.864 m in all cases). We generated 20 total such surrogates for each original trial, or 3,320 of each type of surrogate. Thus, *all* surrogates analyzed (9,660 in total) represented hypothetical walking trials that would have successfully completed the entire trial *without* walking off of the treadmill.

For each surrogate, we then computed a new stride speed (*S_n_*) time series by dividing the surrogate *L_n_* by the surrogate *T_n_* time series. These surrogates were then subjected to the same GEM decomposition and analyses as the original time series. For each trial, the average value of each dependent measure computed across all 20 surrogates for that trial was computed and extracted for statistical analyses.

### Stochastic Control Models of Walking

The stride-to-stride dynamics on the treadmill were modeled as a discrete map:
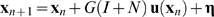
(5)where 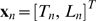 was the state for the current stride *n*, 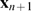 was the corresponding state for the next stride, and 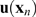 was a vector of control inputs. *I* was the 2×2 identity matrix. *G* was a 2×2 diagonal matrix with diagonal elements *g*_1_ and *g*_2_ denoting additional gains, each set initially to 1 and used *only* as a convenient means to tune the system away from optimality (see Supplementary Text S3). *N* was a 2×2 diagonal multiplicative (i.e., motor output) noise matrix with nonzero diagonal elements. **η** was a 2×1 vector of additive (i.e., sensory and/or perceptual) noise. Non-zero elements of *N* and **η** were taken to be independent, Gaussian random variables with mean zero and standard deviation σ*_k_* (see Supplementary Text S3).

The state update equation (Eq. 5) is intended to model only the discrete-time *inter*-stride walking dynamics. That is, it represents a simple model of the control processes that regulate noise-induced fluctuations away from perfect performance by adjusting *T_n_* and *L_n_*. The choice of states [*T_n_*, *L_n_*] was biologically motivated as these variables are considered *the* fundamental variables of walking (e.g., see [7], [10], [12] and references therein). Together, they form the most basic definition of “walking”: i.e., at each stride, the walker must move a finite distance (*L_n_*) in a finite amount of time (*T_n_*). Overall, we assume walking dynamics are governed by central pattern generator (CPG) processes [71]–[75] yielding repetitive limit cycle behavior [11], [59], [72], [75], [76]. Thus, in the absence of control input and noise, successive strides simply repeat (i.e., **x** *_n_*_+1_ = **x** *_n_*), reflecting the fundamentally cyclical nature of walking. Many suitable differential equation models of such continuous-time walking dynamics exist, ranging from relatively simpler mechanical models [11], [76]–[80] to highly complex neuro-musculo-skeletal models [81]–[84]. A true strength of the approach taken here is that *any* such reasonable model could be used to generate [*T_n_*, *L_n_*] time series. Thus, our results have broad potential impact both for experimental studies of human walking and also for anyone developing computational simulations of walking or actual (physical) walking robots, regardless of their complexity.

The controller was modeled as an unbiased stochastic optimal single-step controller with direct error feedback. This controller design was based on the Minimum Intervention Principle (MIP) [27], [28], but modified to incorporate a preferred operating point (POP) for the controller along the GEM. Accordingly, the cost function took the form:
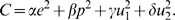
(6)The first term, *αe* ^2^, depended on the definition of the goal-level error for the task [25]. For treadmill walking, we assumed the controller's strategy was to maintain constant speed at each stride, *L_n_*/*T_n_* = *v* (i.e., Eq. 2). Thus, the error the controller sought to minimize was 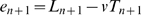 at stride *n*+1. This cost function directly reflects the *strategy* (Eq. 2) we hypothesize subjects adopted to regulate stride variability while satisfying the fundamental task *requirement* defined in Eq. 1. While the underlying task requirement (Eq. 1) does not change, different hypothesized control strategies could be obtained by defining different GEMs (possibly including more and/or different state variables) and would thus change the definition of the error term, *e*, used in the above cost function. The second term in Eq. (6), *βp* ^2^, penalized the distance, *p_n+1_*, of the state at stride *n*+1 from the preferred operating point, [*T* ^*^, *L* ^*^]. The last two terms in Eq. (6) were effort penalty terms where **u** = [*u*_1_, *u*_2_]*^T^* was the control input used to drive the state from stride *n* to stride *n*+1 (Eq. 5). Here, *α*, *β*, *γ*, and *δ* were positive constants that weighted the different components in *C*.

The objective of the controller was to minimize *C* in a probabilistic sense across each trial. That is, we did not minimize the cost itself function directly, but rather its expected value, 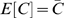. The optimal control inputs *u*_1_ and *u*_2_ were then determined by solving a classic quadratic optimal control problem with an equality constraint. This process yielded optimal control inputs obtained analytically as a function of the current state, **x** *_n_* (see Supplementary Text S3 for details).

The optimal, strictly MIP controller (Fig. 7) was implemented as follows. First, we set *β* = 0 so the cost function, Eq. (6), depended *only* on the goal-level error *e*. This strict MIP controller only corrected *δ_P_* deviations off of the GEM (Fig. 2). When the state, **x** *_n_*,was on the GEM, the controller exerted *no* control effort, since Eq. (6) was already minimized. Since this was true at *all* points along the GEM, the strict MIP controller was neutrally stable along the GEM. Because of the stochastic nature of the trial-to-trial dynamics (Eq. 5), we expected consecutive strides to exhibit random walk behavior (i.e., Brownian motion) along the GEM. Indeed, this was what we obtained in our simulations (Figs. 7E, 7G). We defined a GEM corresponding to a walking speed of *v* = 1.21 m/s, which corresponded to the mean speed of our human subjects walking at 100% of their preferred walking speed (Fig. 3C). To realize the inter-trial dynamics, we then chose the remaining parameter values to approximate the stride speed variability observed in our experimental data (Fig. 3F). For the strict MIP controller, this yielded a stride map, Eq. (5), where *G* = *I* and where the elements of *N* and **η** were defined using σ_1_ = σ_3_ = 0.017 and σ_2_ = σ_4_ = 0.010 (see Supplementary Text S3). For Eq. (6), we set *β* = 0 and *α* = *γ* = *δ* = 10. We note that this strict MIP controller was not able to match the qualitative features of the experimental data (Fig. 7) for any choice of parameter values.

The optimal POP controller (Fig. 8) was implemented as follows. To drive the states to a preferred operating point, [T^*^, L^*^], along the GEM, we set *β* = 2.79 to yield time series that approximated our experimental data. Our results, however, were not sensitive to this value of *β*. This POP controller exerted effort not only perpendicular to the GEM, but also along it. T^*^ was taken to be 1.105s, the mean stride time of our human subjects walking at 100% PWS (Fig. 3B), and L^*^ = vT^*^, where again *v* = 1.21 m/s. All other parameter values for this optimal POP controller were identical to those for the optimal MIP controller. It is important to note that for this POP controller, the *anti*-persistence in the *δ_P_* time-series (Fig. 5D) could not be elicited for any combination of values for the cost function multipliers (*α*, *β*, *γ*, and *δ*) or noise amplitudes 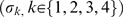.

To match our human data in terms of the anti-persistent DFA exponents in the *δ_P_* time-series (Fig. 5D), we implemented the *sub*-optimal OVC controller (Fig. 9) as follows. This controller was designed to slightly *over*-correct any *δ_P_* deviations away from the GEM. To do this we increased the additional controller gains in *G* from unity to g_1_ = g_2_ = 1.24. We retained the same preferred operating point, [T^*^, L^*^], from the POP controller above (i.e., *β* = 2.79, with *v* = 1.21 m/s, T^*^ = 1.105s and L^*^ = vT^*^), as well as the same weightings for the remaining cost function terms (*α*, *γ*, and *δ*), and the same noise amplitudes 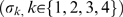. We chose these values to provide a reasonable match to the variability in the *δ_T_* and *δ_P_* directions for the OVC model to the average variability observed in the human (HUM) data (Fig. 9F).

It is important to note that for each model, no explicit or rigorous attempts were made to find “best fits” to our experimental data. For example, we could adjust model parameters to fit different values for the means and SD's of different stride variables to try to more closely replicate the data of any of our individual subjects. However, our overall results were insensitive to the precise parameter values: i.e., the contrasts in the fundamental qualitative features of each of these models will remain the same.

For all three model configurations, we generated 20 simulations of 500 walking strides each to represent a single simulated “average” subject. Model outputs consisted of stride time (*T_n_*) and stride length (*L_n_*) time series. Time series of stride speeds were then calculated as *S_n_* = *L_n_*/*T_n_*, as before. As with our surrogate analyses, we computed the net cumulative distances walked (Eq. 4) by each simulation to ensure no simulation “walked off” the treadmill. Means, standard deviations, and DFA *α* exponents were computed for all primary stride variables (*T_n_*, *L_n_*, and *S_n_*), as we did for the experimental trials. The same GEM decomposition (Eq. 3) was applied to compute *δ_T_* and *δ_P_* deviations along and perpendicular to the GEM. Standard deviations and DFA *α* exponents were then computed for each *δ_T_* and *δ_P_* time series obtained from each simulated walking trial (Figs. 7–9).

### Statistical Analyses

All statistical tests were performed in Minitab 15 (Minitab, Inc., State College, PA). For all dependent measures, we computed between-subject means and ±95% confidence intervals at each walking speed. Where appropriate (Figs. 3, 4C, 6A–B, and 7A–B), linear or quadratic trends across speeds were computed using standard least squares regression [23]. The standard deviations and DFA *α* exponents computed from the experimental (Fig. 5C–D) and surrogate (Figs. 6F–G and 7F–G) data sets were subjected to a 3-factor (Direction×Speed×Subject) mixed-effects, repeated measures, general linear model analysis of variance (ANOVA). Direction (*δ_T_* vs. *δ_P_*) and Speed (80%–120% of PWS) were taken as fixed factors. Subjects (n = 17) was taken as a random factor. There were 2 repeated trials obtained for nearly all subjects and walking speeds (4 total trials were discarded for technical reasons, as stated above). These models tested for main effects for each factor and also for any interaction effects. For the three computational models, the standard deviations and DFA *α* exponents computed from each model (Figs. 7–9, F–G) were subjected to a single-factor (Direction: *δ_T_* vs. *δ_P_*) repeated measures, balanced ANOVA, with 20 repeated observations. For all statistical tests, standard graphical analyses of the model residuals were performed to ensure each test met the linearity and normality assumptions of each ANOVA model.

## Supporting Information

Text S1 Extended description of the construction of Figure 1.
(0.30 MB PDF)

Text S2 Additional surrogate data analyses and results.
(0.44 MB PDF)

Text S3 Derivation of the GEM-based inter-stride optimal controller for treadmill walking.
(0.26 MB PDF)

Text S4 Extended description of the detrended fluctuation analysis algorithm.
(0.27 MB PDF)
