# Supplementary material for: Do Humans Optimally Exploit Redundancy to Control Step Variability in Walking?
Source: PLoS Comput Biol. 2010 Jul 15;6(7):e1000856. doi: 10.1371/journal.pcbi.1000856 (PMC2904769; doi:10.1371/journal.pcbi.1000856)
Supplement: Text S1 — Extended description of the construction of Figure 1. (0.30 MB PDF) [file pcbi.1000856.s001.pdf]

Do Humans Optimally Exploit Redundancy to Control Step Variability in Walking?

Jonathan B. Dingwell, Joby John and Joseph P. Cusumano

PLoS Computational Biology

SUPPLEMENTARY TEXT #S1
Extended Description of the Construction of Figure 1

Zarrugh et al. [85] used experimental data collected during treadmill walking across a wide range of imposed speeds, stride lengths, and stride frequencies to derive detailed expressions for predicting metabolic energy cost as a function of *step* length (*s*) and *step* frequency (*f*). The expression they derived for the energy expenditure per meter walked per kg body mass (combining their Eq. 2 and Eq. 4 from [85]) is:

$$E_m(s,f)=\frac{E_w(s,f)}{v}=\frac{E_0}{s\,f\left(1-s^2/s_u^2\right)\left(1-f^2/f_u^2\right)}=\frac{s_u^2\,f_u^2\,E_0}{s\,f\left(s_u^2-s^2\right)\left(f_u^2-f^2\right)}\,, \tag{7}$$

where *s* is average step length (i.e., $s = L/2$ in meters), *f* is average step *rate* in steps/minute (i.e., $f = (1/T)\times 60$), and E_0, s_u and f_u are all constants that must be derived experimentally for individual subjects [85].

To demonstrate how these contour curves behave, we replicate the results of Zarrugh for one typical subject for whom they obtained experimental values of E_0, s_u and n_u. Fig. 10**A** replicates their Fig. 6b [85], showing energy cost (E_m) as a function of *average step rate* and *average step length*. Fig. 10**B** shows the same energy contours, plotted in terms of *average stride time* and *average stride length* (i.e., [*T*, *L*]) as in the manner of our paper (Fig. 1). In both plots, black diagonal lines indicate the energetically optimal walking speed (in this case, 1.314 m/s) that would indicate the GEM. As shown in Fig. 1 in the main manuscript, near the optimum value, comparable small relative changes in either stride length or stride time result in comparable changes in energy cost.

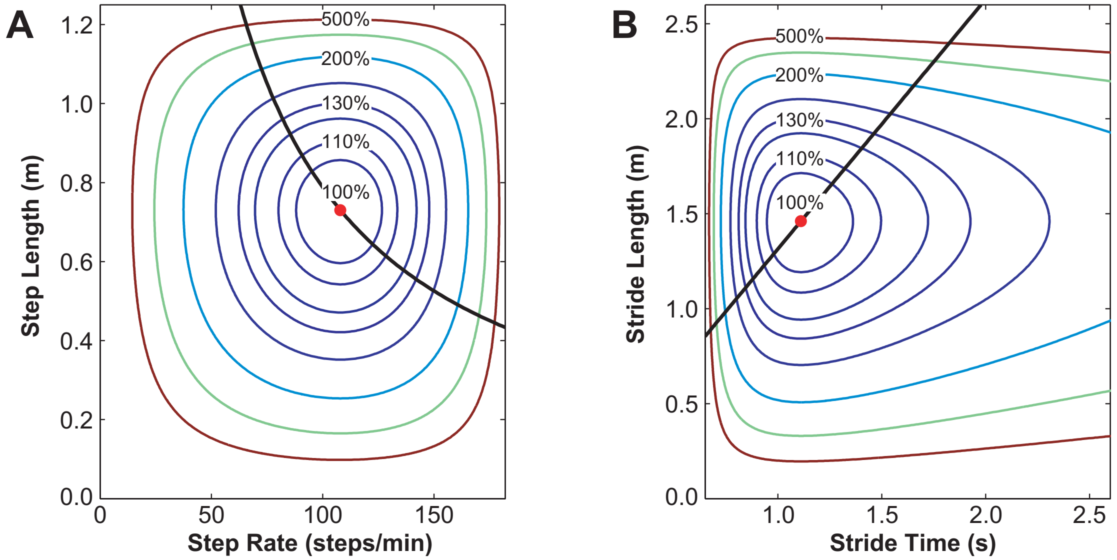

Figure 10 – Energy cost (E_m) contours for a typical subject. Panel (**A**) is plotted in the same manner as Zarrugh et al., 1974 (Ref. [85], their Fig. 6b). Panel (**B**) shows the same data plotted in the manner of Fig. 1 from this manuscript.

References:

85. Zarrugh MY, Todd FN, Ralston HJ (1974) Optimization of Energy Expenditure During Level Walking. European Journal of Applied Physiology 33: 293-306.
