# Supplementary material for: Do Humans Optimally Exploit Redundancy to Control Step Variability in Walking?
Source: PLoS Comput Biol. 2010 Jul 15;6(7):e1000856. doi: 10.1371/journal.pcbi.1000856 (PMC2904769; doi:10.1371/journal.pcbi.1000856)
Supplement: Text S2 — Additional surrogate data analyses and results. (0.44 MB PDF) [file pcbi.1000856.s002.pdf]

Do Humans Optimally Exploit Redundancy to Control Step Variability in Walking?

Jonathan B. Dingwell, Joby John and Joseph P. Cusumano

PLoS Computational Biology

SUPPLEMENTARY TEXT #S2
Additional Surrogate Data Analyses and Results

The independently randomly shuffled surrogates shown in Fig. 6 demonstrate *one* possible viable alternative strategy subjects could have used. Those surrogates represented an alternative strategy that still satisfied the fundamental task requirements (i.e., Eq. 1), but was completely "ignorant" of the proposed GEM, defined by Eq. 2. Here, we present *two* additional alternatives using surrogate data [86,87]. These surrogates also directly tested specific null hypotheses regarding different alternative strategies that made absolutely *no* reference to the GEM that subjects could have used to successfully complete the treadmill walking task (i.e., satisfied Eq. 1).

Independently Phase-Randomized Surrogates:

First, we note that the random shuffling eliminated the statistical persistence observed in humans (Fig. 3**G**,**H**) [88-90]. We therefore tested a second alternative strategy that also regulated T_n and L_n independently of the GEM, but in a way that retained their original statistical persistence (Fig. 11**B**). We implemented this hypothetical controller by generating 20 "*phase-randomized*" surrogates [86,87,91] for each experimental trial. These phase-randomized surrogates were generated separately for the original T_n and L_n time series for each trial by computing the Fourier transform of each original time series, randomizing the phase spectrum portion of the Fourier transform, and then computing the inverse Fourier transform [86,87]. These surrogates tested the null hypothesis that subjects choose stride times and stride lengths that were independent of each other, but that remained temporally correlated across consecutive strides.

All surrogates were constrained so they did *not* "walk off" the treadmill (i.e., so *all* surrogates satisfied Eq. 1). We verified this by computing the maximum forward [max(d_{net})] and minimum backward [min(d_{net})] distances each surrogate walked during the entire trial. In this way, we confirmed that none of the surrogates walked off the treadmill (i.e., min(d_{net}) ≥ -0.864m and max(d_{net}) $\leq +0.864$m for *all* cases). We generated 20 total such surrogates for each original trial. Thus, *all* 3,320 phase-randomized surrogates analyzed represented hypothetical walking trials that would have successfully completed the entire trial *without* walking off of the treadmill. For each surrogate, we then computed a stride speed (S_n) time series by dividing the surrogate L_n by the surrogate T_n time series. These surrogates were then subjected to the same GEM decomposition and analyses as the original experimental time series. For each trial, the average value of each dependent measure computed across all 20 surrogates for that trial was computed and extracted for statistical analyses.

These surrogates preserved the mean, variance (Fig. 11**A**), and probability distribution of each time series just as the randomly shuffled surrogates did. However, the phase-randomized surrogates *also* preserved the power spectra and autocorrelation properties of each original time series [86,87], thus preserving the statistical persistence (Fig. 11**B**) observed in the original T_n and L_n time series (Figs. 3**G**-**H**). Again, by construction, all surrogates satisfied the inequality of Eq. (1) (Fig. 11**C**). However, even though these surrogates preserved the statistical persistence of L_n and T_n (Fig. 11**B**), the statistical anti-persistence observed experimentally for S_n (Fig. 3**I**) was replaced with strong statistical persistence (Fig. 11**B**).

These phase randomized surrogates also exhibited approximately isotropic distributions about [T^*, L^*] in the [T_n, L_n] plane (Fig. 11**D**). The δ_P and δ_T times series were also qualitatively very similar to each other (Fig. 11**E**). Standard deviations for δ_P and δ_T (Fig. 11**F**) were both much closer to 1 than the originals, but remained significantly different from each other ($F_{(1,16)} = 39.525$; $p = 1.08 \times 10^{-5}$). DFA exponents for δ_P and δ_T (Fig. 11**G**) both exhibited $\alpha >> ½$, but remained significantly different from each other ($F_{(1,16)} = 73.222$; $p = 2.29 \times 10^{-7}$).

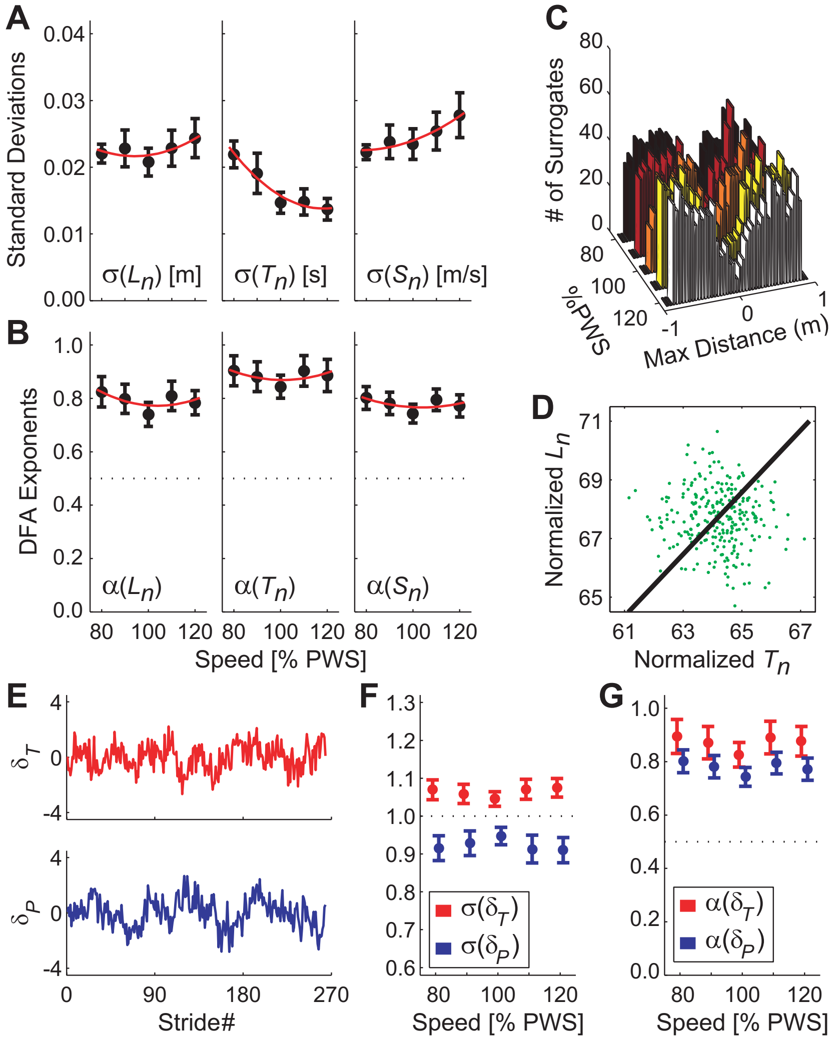

Figure 11 – Phase-Randomized Surrogate Walking. All error bars represent between-subject ±95% confidence intervals. By definition, these surrogates exhibited the same mean stride parameters (not shown) as the original walking data (Fig. 3**A**-**C**).
(A) The variability of stride length (L_n) and stride time (T_n) were the same as for the original data (Fig. 3**D**-**E**), while the variability of stride speed (S_n) was slightly increased (Fig. 3**F**).
(B) The statistical persistence for both L_n and T_n was also the same as the experimental trials (Fig. 3**G**-**H**). However, unlike humans (Fig. 3**I**), these surrogates exhibited strong statistical persistence ($\alpha >> ½$) for stride speed (S_n).
(C) Histograms of maximum forward and backward distances walked by all surrogates. No surrogate walked beyond either the front or back edges of the treadmill belt (± 0.864 m).
(D) A typical surrogate for the trial shown in Fig. 5**A**. The GEM remains the same. However, the distribution of strides is now nearly isotropic.
(E) Time series of δ_T and δ_P deviations for the surrogate trial shown in (**D**). In this case, both time series exhibit statistical persistence.
(F) Variability (σ) was greater for δ_T deviations than for δ_P deviations ($F_{(1,16)} = 39.525$; $p = 1.08 \times 10^{-5}$). However, this difference was much less pronounced than for the original data (Fig. 5**C**).
(G) DFA exponents (α) were significantly greater for δ_T deviations than for δ_P deviations ($F_{(1,16)} = 73.222$; $p = 2.29 \times 10^{-7}$). However, unlike humans (Fig. 5**D**), these surrogates did not exhibit anti-persistent δ_P fluctuations, but instead exhibited strong statistical persistence ($\alpha >> ½$) for δ_P deviations.

Most importantly, these surrogates exhibited drastically different dynamics from humans (Fig. 5). Thus, the null hypothesis that subjects used this second alternative strategy was also rejected.

Paired Randomly Shuffled Surrogates:

A third possibility is that the covariation observed in [T_n, L_n] (Figs. 5**A**,**C**) was *not* due to stride-to-stride "control," but rather to simple biomechanics [92]: i.e., taking longer (or shorter) strides (increased or decreased L_n) naturally produced slower (or faster) strides (increased or decreased T_n). To test this, we generated 20 "*paired"* randomly shuffled surrogates for each experimental trial. We kept all the original [T_n, L_n] pairings unchanged, but then shuffled these matched pairs in random temporal order. By construction, therefore, these surrogates exhibited the exact same [T_n, L_n] covariation as the original data. However, this hypothesized control strategy was still "GEM-ignorant" because each stride was chosen independently, with no explicit stride-to-stride *control*. Thus, these surrogates tested the null hypothesis that stride times (T_n) and stride lengths (L_n) may have been coupled mechanically, but were still chosen independently from each stride to the next.

Again, we confirmed that none of the surrogates walked off the treadmill (i.e., $\min(d_{net}) \geq -0.864$m and $\max(d_{net}) \leq +0.864$m in all cases). We generated 20 total such surrogates for each original trial. Thus, *all* 3,320 of these paired randomly shuffled surrogates also represented hypothetical walking trials that would have successfully completed the entire trial *without* walking off of the treadmill. For each surrogate, we then computed a stride speed (S_n) time series by dividing the surrogate L_n by the surrogate T_n time series. These surrogates were then subjected to the same GEM decomposition and analyses as the original experimental time series. For each trial,

Figure 12 – Paired Randomly Shuffled Surrogate Walking. All error bars represent between-subject ±95% confidence intervals. By definition, these surrogates exhibited the same mean stride parameters (not shown) as the original walking data (Fig. 3**A-C**).
(**A**) These surrogates exhibited the same L_n, T_n, and S_n variability as the original data (Fig. 3**D-F**).
(**B**) Unlike humans (Fig. 3**G-I**), these surrogates exhibited no temporal correlations (all $\alpha \approx ½$) for any of the basic stride parameters (Note, the vertical scale is *very* different from Fig. 3**G-I**)!
(**C**) Histograms of maximum forward and backward distances walked by all surrogates. No surrogate walked beyond either the front or back edges of the treadmill belt (± 0.864 m).
(**D**) A typical surrogate for the trial shown in Fig. 5**A**. Both the GEM and the distribution of strides around the GEM remain identical to Fig. 5**A**.
(**E**) Time series of δ_T and δ_P deviations for the surrogate trial shown in (**D**). In this case, neither time series exhibited statistical persistence.
(**F**) Standard deviations (σ) for both δ_T and δ_P time series. By definition, these were exactly the same as for human subjects (Fig. 5**C**).
(**G**) DFA α exponents for δ_T and δ_P time series at all 5 walking speeds. *Unlike* the experimental trials, there were *no* strong temporal correlations ($\alpha \approx ½$) (Compare to Fig. 5**D** and note the different vertical scales.

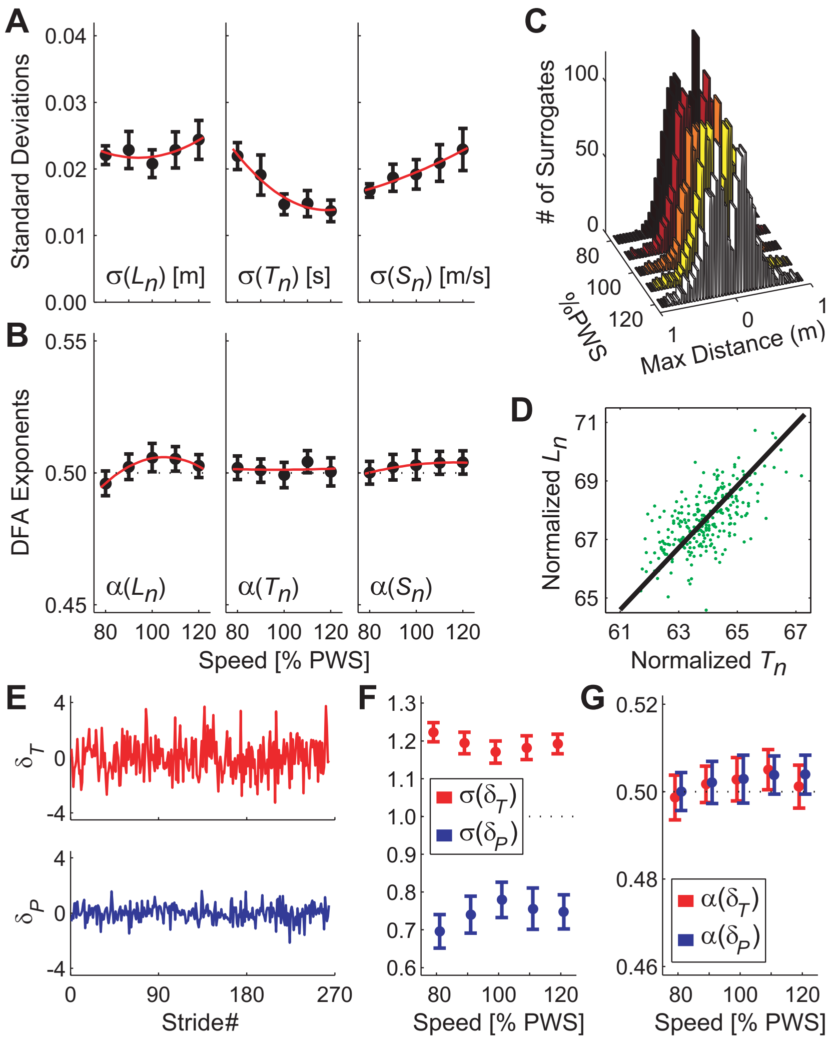

the average value of each dependent measure computed across all 20 surrogates for that trial was computed and extracted for statistical analyses.

By construction, these surrogates preserved the exact same mean, variance (Fig. 12**A**), and probability distribution of each time series. They also satisfied the inequality of Eq. (1) (Fig. 12**C**). Most importantly, these paired surrogates also retained the exact same covariation between T_n and L_n as the original data (Fig. 12**D**, **F**). Hence, these paired randomly shuffled surrogates exhibited the exact same *an*isotropic distributions about $[T^*, L^*]$ in the $[T_n, L_n]$ plane (i.e., Fig. 12**D** is identical to Fig. 5**A**, by definition). As a result, the standard deviations in δ_P and δ_T also remained the same as the original data (i.e., Fig. 12**F** is identical to Fig. 5C).

However, by eliminating the temporal sequencing, all the relevant time series, including L_n, T_n, and S_n (Fig. 12**B**) and δ_P and δ_T (Figs. 12**E** and 12**G**) exhibited $\alpha \approx ½$. Thus, even though these paired randomly shuffled surrogates retained the exact same *variance* structure as the original data sets, the stride-to-stride *dynamics* are clearly completely different. Therefore, the null hypothesis that accounting for (possibly biomechanical) co-variation between L_n and T_n alone might explain our experimental results was also rejected.

<u>*Summary and Discussion:*</u>

The central purpose of these surrogate analyses is to demonstrate that there exists a range of viable strategies that our human subjects *could* have used to satisfy the fundamental walking task constraint defined by Eq. 1 *without* making explicit reference to the hypothesized GEM proposed in Eq. 2. Each surrogate can therefore be thought of as one possible candidate model for describing how humans accomplish this treadmill walking task.

All 3 sets of surrogates presented here (Figs. 6, 11, and 12) fully satisfied Eq. 1, but also exhibited stride-to-stride dynamics that were significantly and qualitatively *different* from humans. Therefore, subjects clearly *could* have successfully accomplished the prescribed walking task (Eq. 1) using control strategies completely ignorant of the GEM's existence, but they did *not* do this.

To be certain, there are *many* more other possible surrogates that one could construct that would test different null hypotheses about how this treadmill walking task might have been accomplished. Each such surrogate would similarly represent another new and different data-based model of how people controlled their stride-to-stride movements. Indeed, the basic paradigm of doing such surrogate analyses strongly encourages researchers to test a *range* of candidate surrogates to ultimately determine an appropriate model structure for a specific experimental data set [86,87]. However, such surrogate testing is only an indirect substitute for developing direct, mathematical control models of these processes, similar to the ones we present in Figs. 7-9.

The surrogates presented here demonstrate three concrete results. First, stride-to-stride variations in these most basic of gait parameters are not simply uncorrelated random fluctuations (Fig. 6). Second, accounting for temporal correlations in T_n and L_n independently (e.g., as done in [88-90]) does not sufficiently describe the control dynamics (Fig. 11). Third, accounting for coupling between T_n and L_n is also not sufficient to capture the observed experimental walking dynamics (Fig. 12). In particular, the paired surrogates (Fig. 12) demonstrate quite clearly that quantifying variance ratios alone (as done in applications of UCM and MIP) can very easily lead to incorrect conclusions about control [92]. Our results demonstrate that it is critical to quantify both variability *and* temporal dynamics [93,94] to fully determine how such repetitive movements are controlled.

References Cited:

86. Theiler J, Eubank S, Longtin A, Galdrikian B, Farmer JD (1992) Testing for Nonlinearity in Time Series: the Method of Surrogate Data. Physica D: Nonlinear Phenomena 58: 77-94.

87. Schreiber T, Schmitz A (2000) Surrogate Time Series. Physica D: Nonlinear Phenomena 142: 346-382.

88. Hausdorff JM, Mitchell SL, Firtion R, Peng CK, Cudkowicz ME, et al. (1997) Altered Fractal Dynamics of Gait: Reduced Stride Interval Correlations with Aging and Huntington's Disease. Journal of Applied Physiology 82: 262-269.

89. Hausdorff JM, Peng CK, Ladin Z, Wei JY, Goldberger AL (1995) Is Walking a Random Walk? Evidence for Long-Range Correlations in Stride Interval of Human Gait. Journal of Applied Physiology 78: 349-358.

90. Gates DH, Dingwell JB (2007) Peripheral Neuropathy Does Not Alter the Fractal Dynamics of Gait Stride Intervals. Journal of Applied Physiology 102: 965-971.

91. Dingwell JB, Cusumano JP (2000) Nonlinear Time Series Analysis of Normal and Pathological Human Walking. Chaos: An Interdisciplinary Journal of Nonlinear Science 10: 848-863.

92. Valero-Cuevas FJ, Venkadesan M, Todorov E (2009) Structured variability of muscle activations supports the minimal intervention principle of motor control. Journal of Neurophysiology 102: 59-68.

93. Gates DH, Dingwell JB (2008) The Effects of Neuromuscular Fatigue on Task Performance During Repetitive Goal-Directed Movements. Experimental Brain Research 187: 573-585.

94. Gates DH, Su JL, Dingwell JB (2007) Possible biomechanical origins of the long-range correlations in stride intervals of walking. Physica A: Statistical and Theoretical Physics 380: 259-270.
