# Supplementary material for: Do Humans Optimally Exploit Redundancy to Control Step Variability in Walking?
Source: PLoS Comput Biol. 2010 Jul 15;6(7):e1000856. doi: 10.1371/journal.pcbi.1000856 (PMC2904769; doi:10.1371/journal.pcbi.1000856)
Supplement: Text S3 — Derivation of the GEM-based inter-stride optimal controller for treadmill walking. (0.26 MB PDF) [file pcbi.1000856.s003.pdf]

Do Humans Optimally Exploit Redundancy to Control Step Variability in Walking?

Jonathan B. Dingwell, Joby John and Joseph P. Cusumano

PLoS Computational Biology

SUPPLEMENTARY TEXT #S3
Derivation of the GEM-Based Inter-Stride Optimal Controller for Treadmill Walking

---

We start by defining the goal function F:

$$F = L_n - vT_n , \tag{8}$$

which, when set equal to zero, gives a specific hypothesis on the strategy (see Eq. 2) used by subjects to carry out the treadmill walking task (Eq. 1). That is, when $F = 0$ the task is executed perfectly, in that at stride n the speed, L_n/T_n, exactly equals v, the treadmill speed. Thus, deviations away from zero in the value of the goal function represent the error in the task execution. We also define the distance D of the current state $[T_n, L_n]$ from $[T^*, L^*]$, a preferred operating point (POP), as:

$$D = \sqrt{(T_n - T^*)^2 + (L_n - L^*)^2} . \tag{9}$$

Thus, $D = 0$ corresponds to walking exactly at the POP.

The inter-stride dynamics for the task is modeled as an update map with controllers $u_1(T_n, L_n)$ and $u_2(T_n, L_n)$ as:

$$T_{n+1} = T_n + g_1(1 + \sigma_1\nu_1)u_1 + \sigma_3\nu_3 \tag{10a}$$

$$L_{n+1} = L_n + g_2(1 + \sigma_2\nu_2)u_2 + \sigma_4\nu_4 \tag{10b}$$

where: the stride time-length pair $[T_n, L_n]$ serves as the state variable; g_1 and g_2 are gains; the ν_k $(k \in \{1,2,3,4\})$ are independent random variables with zero mean and unit variance; and the σ_k give the standard deviations of each noise term. We note that Eq. (10) can be put into vector form as (see also Eq. 5):

$$\mathbf{x}_{n+1} = \mathbf{x}_n + G(I + N)\,\mathbf{u}(\mathbf{x}_n) + \boldsymbol{\eta} , \tag{11}$$

where $\mathbf{x}_n = [T_n, L_n]^T$, $\boldsymbol{\eta} = [\sigma_3\nu_3, \sigma_4\nu_4]^T$, I is the 2×2 identity matrix, and the diagonal noise and gain matrices N and G are given by

$$N = \begin{bmatrix} \sigma_1\nu_1 & 0 \\ 0 & \sigma_2\nu_2 \end{bmatrix} \quad \text{and} \quad G = \begin{bmatrix} g_1 & 0 \\ 0 & g_2 \end{bmatrix}, \tag{12}$$

respectively.

It is important to emphasize that the above controller is only intended to model the *inter*-stride dynamics, not the full neuro-biomechanical dynamics *within* each walking stride. That is, it represents a simple model of the neuro-motor control processes that regulate fluctuations away from perfect performance by adjusting the stride time (T_n) and length (L_n), which should be thought of as *control parameters* for the neuro-biomechanical action required to generate each individual step. Thus, our model implies an overall hierarchical control of locomotion in which the *inter*-stride controller of Eq. (10) provides error correction to an *intra*-stride controller that is approximately "ballistic" or "feed-forward" during each individual step. While real humans certainly apply continuous on-line control within each stride, there is no loss of generality here because the discrete *inter*-stride

controller of Eq. (10) can be thought of as representing the summed or *net* effect of this on-line control, enacted over one complete gait cycle.

We find the control inputs u_1 and u_2 by solving a classic quadratic optimal control problem with an equality constraint (see e.g., [95-98]). The controllers are chosen to be optimal with respect to the expected value of a cost function with the general form:

$$C = \alpha e^2 + \beta p^2 + \gamma u_1^2 + \delta u_2^2 , \tag{13}$$

in which e represents the error in the stride-speed (i.e., the deviation of F from zero), and p represents the distance from the POP (i.e., the deviation of D from zero), both at step $n + 1$. As described below, the constraint arises because we further specify our model by requiring u_1 and u_2 to be single step, unbiased controllers that seek to drive Eq. (8) to zero at each stride.

The form of Eq. (13) is sufficiently general to include all cases studied in this work. Specifically, when $\beta = 0$, the resulting controller corresponds to the ideal Minimum Intervention Principle (MIP) case, whereas for $\beta > 0$ the system contains a preferred operating point (POP). Furthermore, the gains in Eq. (10) are initially both set to $g_1 = g_2 = 1$ to find the *optimal* control inputs, as in what follows. Then, given these optimal inputs, which, as we show below are linear in the state $[T_n, L_n]$, we can then use non-unity gains as a simple means to tune the control away from optimality for the overcorrecting (OVC) case.

Given the above structure, the first step in finding the controllers is to substitute for e and p using F and D from Eqs. (8) and (9), respectively, into Eq. (13):

$$C = \alpha \left(L_{n+1} - vT_{n+1}\right)^2 + \beta\left[\left(T_{n+1} - T^*\right)^2 + \left(L_{n+1} - L^*\right)^2\right] + \gamma u_1^2 + \delta u_2^2 , \tag{14}$$

Then, T_{n+1} and L_{n+1} are replaced with the right hand sides of the map defined by Eqs. (10) to give:

$$\begin{aligned} C = {} & \alpha\left\{\left[L_n + (1+\sigma_2\nu_2)u_2 + \sigma_4\nu_4\right] - v\left[T_n + (1+\sigma_1\nu_1)u_1 + \sigma_3\nu_3\right]\right\}^2 + \\ & \beta\left\{\left[T_n + (1+\sigma_1\nu_1)u_1 + \sigma_3\nu_3 - T^*\right]^2 + \left[L_n + (1+\sigma_2\nu_2)u_2 + \sigma_4\nu_4 - L^*\right]^2\right\} + \gamma u_1^2 + \delta u_2^2 . \end{aligned} \tag{15}$$

We next expand Eq. (15) and take its expected value, $\overline{C} = E[C]$, using the fact that the noise processes have zero mean, unit variance, and are uncorrelated. That is, we set $E[\nu_k^2] = 1$ and $E[\nu_k] = 0$ for $k \in \{1,2,3,4\}$, and $E[\nu_k \nu_m] = 0$ for $k \neq m$. This gives:

$$\begin{aligned} \overline{C} = {} & 2u_1\left(\alpha T_n v^2 - \alpha L_n v - \beta T^* + \beta T_n\right) + u_1^2\left[\left(\sigma_1^2 + 1\right)\alpha v^2 + \left(\sigma_1^2 + 1\right)\beta + \gamma\right] + \left(\alpha T_n^2 + \sigma_3^2\alpha\right)v^2 - \\ & 2u_2\left[\alpha T_n v + \beta L^* - (\alpha + \beta)L_n\right] - 2\alpha u_1 u_2 v - 2\alpha L_n T_n v + u_2^2\left[\delta + \left(\sigma_2^2 + 1\right)\alpha + \left(\sigma_2^2 + 1\right)\beta\right] + \\ & \beta T^{*2} - 2\beta T_n T^* + \beta L^{*2} - 2\beta L_n L^* + \beta T_n^2 + (\alpha + \beta)L_n^2 + \sigma_4^2\alpha + \left(\sigma_3^2 + \sigma_4^2\right)\beta . \end{aligned} \tag{16}$$

The minimum of $\overline{C}$ occurred when the state was exactly on the GEM ($e = 0$) and (if $\beta > 0$) at the preferred operating point ($p = 0$), so that the control efforts u_1 and u_2 were also zero. The two noise terms in Eq. (11), N and $\boldsymbol{\eta}$, however, had the effect that the state, $\mathbf{x}_n$, never completely converged onto the GEM. At each stride, this controller attempted to correct the error from one previous stride only.

The requirement that the single-step controller be unbiased means that it is perfect *on average*, so that the expected value of the goal function F (i.e., the error) at the next step, n+1, is zero:

$$\overline{F} \equiv E\left[L_{n+1} - vT_{n+1}\right] = 0 . \tag{17}$$

Substituting for $[T_{n+1}, L_{n+1}]$ in F using Eqs. (10) and taking the expected value thus yields a constraint $\overline{F} = E[F]$ on the controller dynamics as

$$\overline{F} = -(u_1 + T_n)v + u_2 + L_n \,. \tag{18}$$

We then form the augmented Lagrangian, Λ, as [95-98]:

$$\Lambda = \overline{C} - \mu \overline{F} \,. \tag{19}$$

where μ is a Lagrange multiplier. The optimal controllers extremize Λ. We thus differentiate the augmented Lagrangian and set the resulting expressions equal to zero to form the system of algebraic equations:

$$\frac{\partial \overline{C}}{\partial u_i} - \mu \frac{\partial \overline{F}}{\partial u_i} = 0, \qquad i = 1, 2 \cdot \tag{20}$$

After making appropriate substitutions for $\overline{C}$ and $\overline{F}$ and reducing, these expressions yield:

$$2\left[(\sigma_2^2 + 1)\alpha + (\sigma_2^2 + 1)\beta + \delta\right]u_2 - 2\alpha u_1 v - 2\alpha T_n v - 2\beta L^* + 2(\alpha + \beta)L_n - \mu = 0 \tag{21a}$$

$$2\left[(\sigma_1^2 + 1)\alpha v^2 + (\sigma_1^2 + 1)\beta + \gamma\right]u_1 - 2\alpha u_2 v + 2\alpha T_n v^2 - 2\beta T^* + 2\beta T_n + (\mu - 2\alpha L_n)v = 0 \tag{21b}$$

Using the above equations, together with the constraint of Eq. (18) allows one to solve for u_1 and u_2. (One can also solve for the Lagrange multiplier, μ, however we do not use it here.)

The optimal controllers obtained via the above procedure were found to be:

$$u_1 = \frac{-T_n\left[(\delta + \sigma_2^2\,\alpha + (\sigma_2^2 + 1)\beta)v^2 + \beta\right] + L_n v\left[\delta + \sigma_2^2(\alpha + \beta)\right] + \beta\left(L^* v + T^*\right)}{\left[\delta + (\sigma_1^2 + \sigma_2^2)\alpha + (\sigma_2^2 + 1)\beta\right] v^2 + (\sigma_1^2 + 1)\beta + \gamma} \tag{22a}$$

$$u_2 = \frac{-L_n\left[\gamma + (\sigma_1^2\,\alpha + \beta)v^2 + (\sigma_1^2 + 1)\beta\right] + T_n v\left[\gamma + \sigma_1^2(\alpha v^2 + \beta)\right] + \beta v\left(L^* v + T^*\right)}{\left[\delta + (\sigma_1^2 + \sigma_2^2)\alpha + (\sigma_2^2 + 1)\beta\right] v^2 + (\sigma_1^2 + 1)\beta + \gamma} \tag{22b}$$

Examination of the above result indicates that in this case the controllers, due to the linearity of the goal function Eq. (8), are linear in the state $[T_n, L_n]$. In fact, for both the MIP and POP controllers, by incorporating Eqs. (22) into Eq. (10) the vector form of the update map Eq. (11) can be put into the form:

$$\mathbf{y}_{n+1} = \mathbf{y}_n + (I + N)GA\mathbf{y}_n + \boldsymbol{\eta}, \tag{23}$$

where for control inputs $\mathbf{u} = [u_1, u_2]^T$, A is a 2×2 matrix defined by $\mathbf{u} = A\mathbf{y}_n$ and $\mathbf{y}_n$ is the appropriate state at stride n. For the $\beta = 0$ case, the state is $\mathbf{y}_n = [T_n, L_n]^T = \mathbf{x}_n$, whereas for $\beta > 0$, $\mathbf{y}_n = [T_n - T^*, L_n - L^*]^T$. Thus, since the optimal matrix A is derived with the gain matrix set equal to the identity matrix, $G = I$, it is clear that for $G \neq I$, the *effective* controller matrix, GA, is sub-optimal with respect to the cost function Eq. (13).

References Cited:

95. Luenberger DG, Ye Y (2008) Linear and Nonlinear Programming. New York: Springer Science+Business Media.

96. Kirk DE (2004) Optimal Control Theory: An Introduction. Mineola, NY: Dover Publications.

97. Ogata K (1995) Discrete-Time Control Systems. Upper Saddle River, NJ: Prentice Hall.

98. Stengel RF (1994) Optimal Estimation and Control. Mineola, NY: Dover Publications.
