# Supplementary material for: Do Humans Optimally Exploit Redundancy to Control Step Variability in Walking?
Source: PLoS Comput Biol. 2010 Jul 15;6(7):e1000856. doi: 10.1371/journal.pcbi.1000856 (PMC2904769; doi:10.1371/journal.pcbi.1000856)
Supplement: Text S4 — Extended description of the detrended fluctuation analysis algorithm. (0.27 MB PDF) [file pcbi.1000856.s004.pdf]

Do Humans Optimally Exploit Redundancy to Control Step Variability in Walking?

Jonathan B. Dingwell, Joby John and Joseph P. Cusumano

PLoS Computational Biology

SUPPLEMENTARY TEXT #S4
Extended Description of the Detrended Fluctuation Analysis Algorithm

Detrended Fluctuation Analysis (DFA; Fig. 13) [99-102] was used to determine the degree to which each time series exhibited persistent or anti-persistent temporal correlations across successive strides. DFA has been used extensively to analyze physiological time series because it is robust to noise and nonstationarities [100]. Complete details of the DFA algorithm are published elsewhere [99-102].

In brief, each time series $x(n)$, where $n \in \{1, \dots, N\}$ strides, was first integrated to form a cumulative sum:

$$y(k) = \sum_{i=1}^{k} [x(n) - \bar{x}] \tag{24}$$

where $x(n)$ was the value of x for the nth stride and $\bar{x}$ was the mean value of x across all N strides. Each integrated series was divided into equal, non-overlapping segments of length j. Each segment was detrended by subtracting a least squares linear fit to that segment (Fig. 13B). The squares of the residuals were then averaged over the entire data set and the square root of the mean residual, $F(j)$, was calculated:

$$F(j) = \sqrt{\frac{1}{N} \sum_{k=1}^{N} [y(k) - y_{fit}(k)]^2} \tag{25}$$

This process was repeated for different segment lengths, j. Fifty values of j that were evenly distributed between 4 and $N/4$ were used. Typically, $F(j)$ increases with j and a graph of $\log[F(j)]$ versus $\log(j)$ will exhibit a power-law relationship indicating the presence of scaling (Fig. 13**C**), such that $F(j) \approx j^{\alpha}$ [99-101]. These $\log[F(j)]$ versus $\log(j)$ plots were then fit with a linear function using least squares regression. The slope of this line defined the scaling exponent α (Fig. 13**C**). A value of $\alpha = 0.5$ indicates that the time series is uncorrelated white noise. When $\alpha < 0.5$, the time series contains anti-persistent temporal correlations. When $\alpha > 0.5$, persistent temporal correlations exist [99,100]. When $\alpha = 1.5$, the time series is brown noise (i.e., integrated white noise) [99,100]. Although one could alternatively perform higher order (e.g., quadratic) detrending, this did not alter results obtained from similar time series [103,104].

References:

99. Peng C-K, Buldyrev SV, Goldberger AL, Havlin S, Sciortino F, et al. (1992) Long-Range Correlations in Nucleotide Sequences. Nature 356: 168-170.

100. Hausdorff JM, Peng CK, Ladin Z, Wei JY, Goldberger AL (1995) Is Walking a Random Walk? Evidence for Long-Range Correlations in Stride Interval of Human Gait. Journal of Applied Physiology 78: 349-358.

101. Peng C-K, Buldyrev SV, Hausdorff JM, Havlin S, Mietus JE, et al. (1994) Non-Equilibrium Dynamics as an Indispensable Characteristic of a Healthy Biological System. Integrative Physiological and Behavioral Science 29: 283-293.

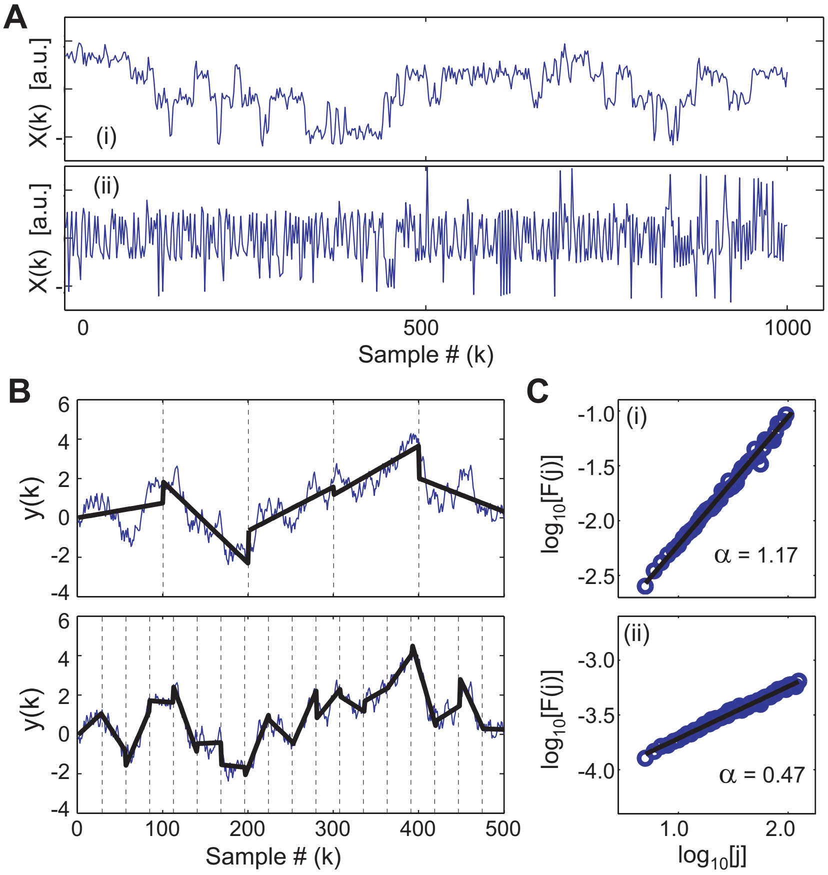

Figure 13 – Illustration of detrended fluctuation analysis.
(**A**) Examples of two simulated stochastic time series, $x(k)$, in arbitrary units (a.u.) with equal variance but very different temporal correlation structures. The top graph (i) clearly shows temporal persistence, whereas the bottom graph (ii) fluctuates much more rapidly.
(**B**) Each time series was first integrated (thin blue lines, Eq. S5-1) and then divided into bins, where each bin contains j samples. Data within each bin were fitted with a straight line (thick black lines) using linear regression. This linear fit was then subtracted from the original and the root mean square of the residuals was calculated (Eq. S5-2). This process was repeated for a range of bins lengths between 4 and 125. As bin size decreased the error also decreased.
(**C**) DFA analyses of the two corresponding time series shown in **A**. The different temporal correlation patterns are clearly reflected in the different scaling exponent values obtained (α = 1.17 vs. α = 0.47). Thus, the α exponent obtained from DFA quantifies temporal correlations (persistence vs. anti-persistence) in a time series, independent of the magnitude of the variance.

102. Goldberger AL, Amaral LAN, Hausdorff JM, Ivanov PC, Peng C-K, et al. (2002) Fractal Dynamics in Physiology: Alterations with Disease and Aging. Proceedings of the National Academy of Sciences USA 99: 2466-2472.

103. Gates DH, Dingwell JB (2007) Peripheral Neuropathy Does Not Alter the Fractal Dynamics of Gait Stride Intervals. Journal of Applied Physiology 102: 965-971.

104. Gates DH, Su JL, Dingwell JB (2007) Possible biomechanical origins of the long-range correlations in stride intervals of walking. Physica A: Statistical and Theoretical Physics 380: 259-270.
